# Supplementary material for: Proteomics reveals signal peptide features determining the client specificity in human TRAP-dependent ER protein import
Source: Nat Commun. 2018 Sep 14;9:3765. doi: 10.1038/s41467-018-06188-z (PMC6138672; doi:10.1038/s41467-018-06188-z)
Supplement: Supplementary file 1 — Supplementary Information [file 41467_2018_6188_MOESM1_ESM.pdf]

Supplementary Information for

Proteomics reveals signal peptide features determining the client specificity in human TRAP-dependent ER protein import

Nguyen *et al.*

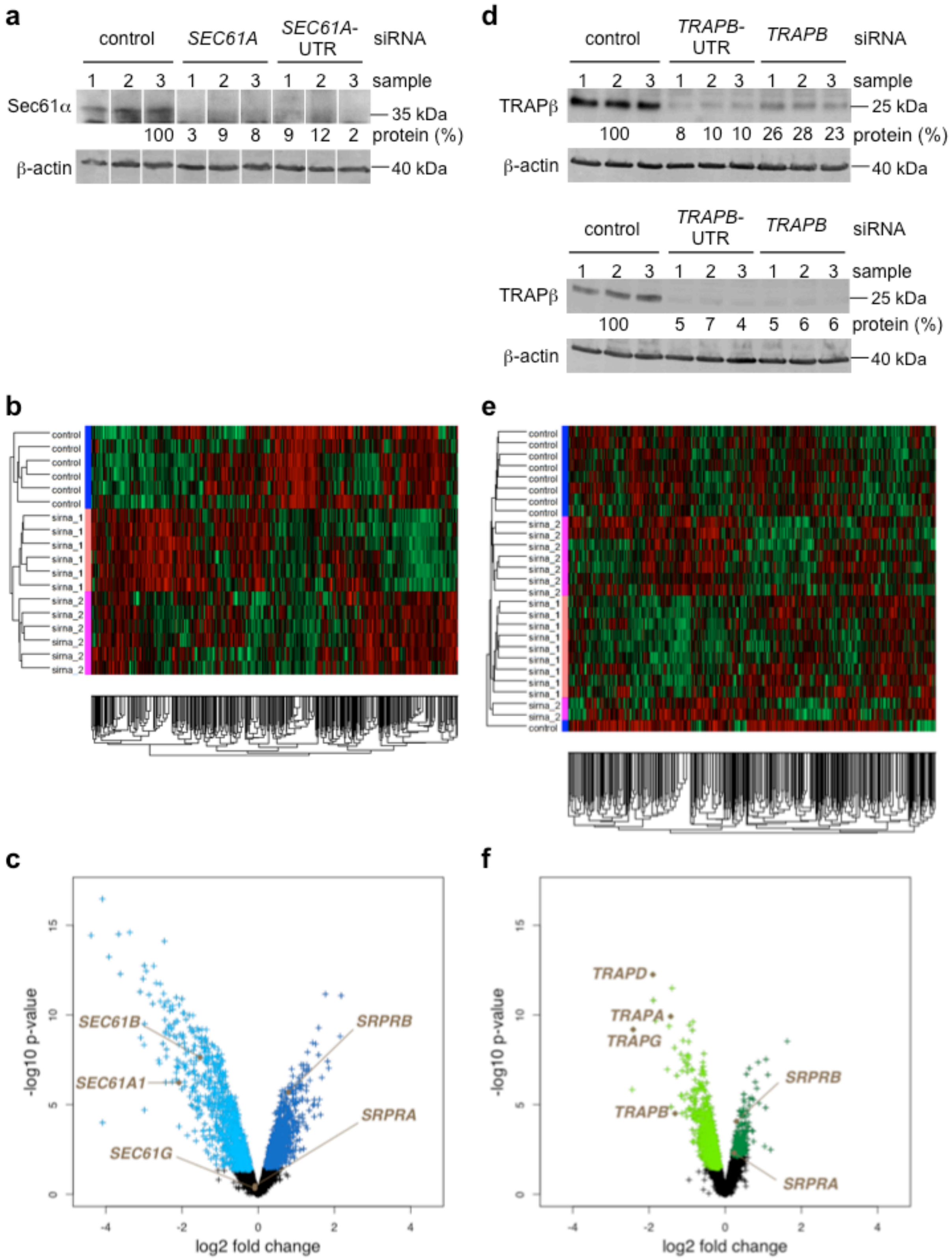

Supplementary Figure 1 | Identification of TRAP clients and compensatory proteins by Sec61 or TRAP depletion in HeLa cells. The color coding follows Fig. 1. (**a** and **d**) The knock-down efficiencies in experiments 2 and 3 were evaluated by western blot analysis and

are presented as the percentage of residual protein levels relative to control. (**b** and **e**) Heat maps visualize clusters of proteins that were significantly upregulated following treatment with both siRNAs directed against either *SEC61A1* or *TRAPB* mRNA or with non-targeting (control) siRNA, or that were significantly downregulated following treatment with both siRNAs, or that represent variations between siRNAs. Red indicates positively affected proteins, green indicates negatively affected proteins. *SEC61A1* siRNA is referred to as sirna1, *SEC61A1-UTR* siRNA as sirna2, *TRAPB-UTR* siRNA as sirna1, *TRAPB* siRNA as sirna2. (**c** and **f**) Differentially affected proteins were characterized by the mean difference of their intensities plotted against the respective permutation false discovery rate-adjusted *P* values in volcano plots. The results for a single siRNA are shown in each case (*SEC61A1* siRNA, *TRAPB-UTR* siRNA). Subunits of the Sec61- and TRAP-complexes and of the SRP receptor are indicated.

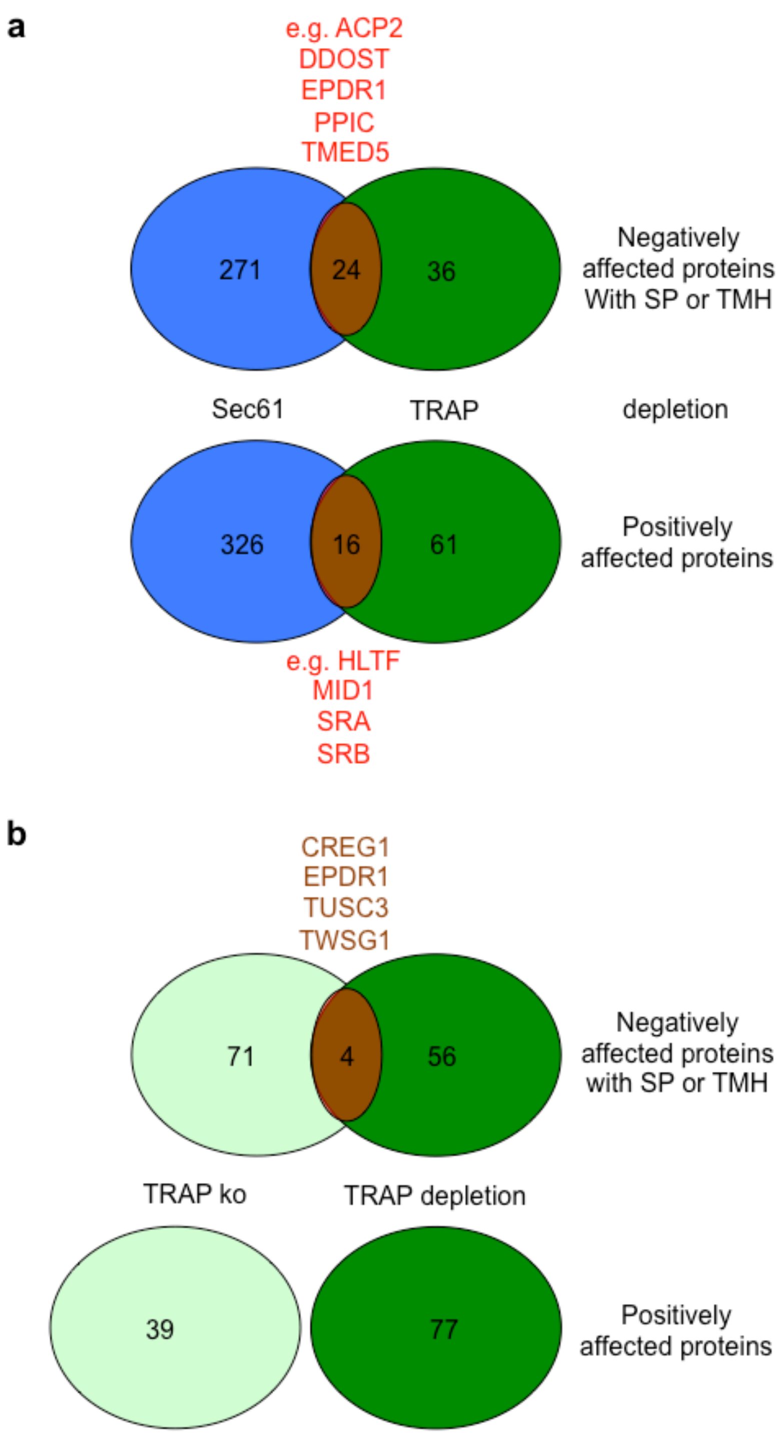

Supplementary Figure 2 | Venn diagrams for negatively and positively affected proteins. (**a**) Comparison between Sec61 and TRAP depletion. (**b**) Comparison between CDG patient fibroblasts (TRAP ko) and TRAP depletion. Only negatively affected with SP or TMH are shown.

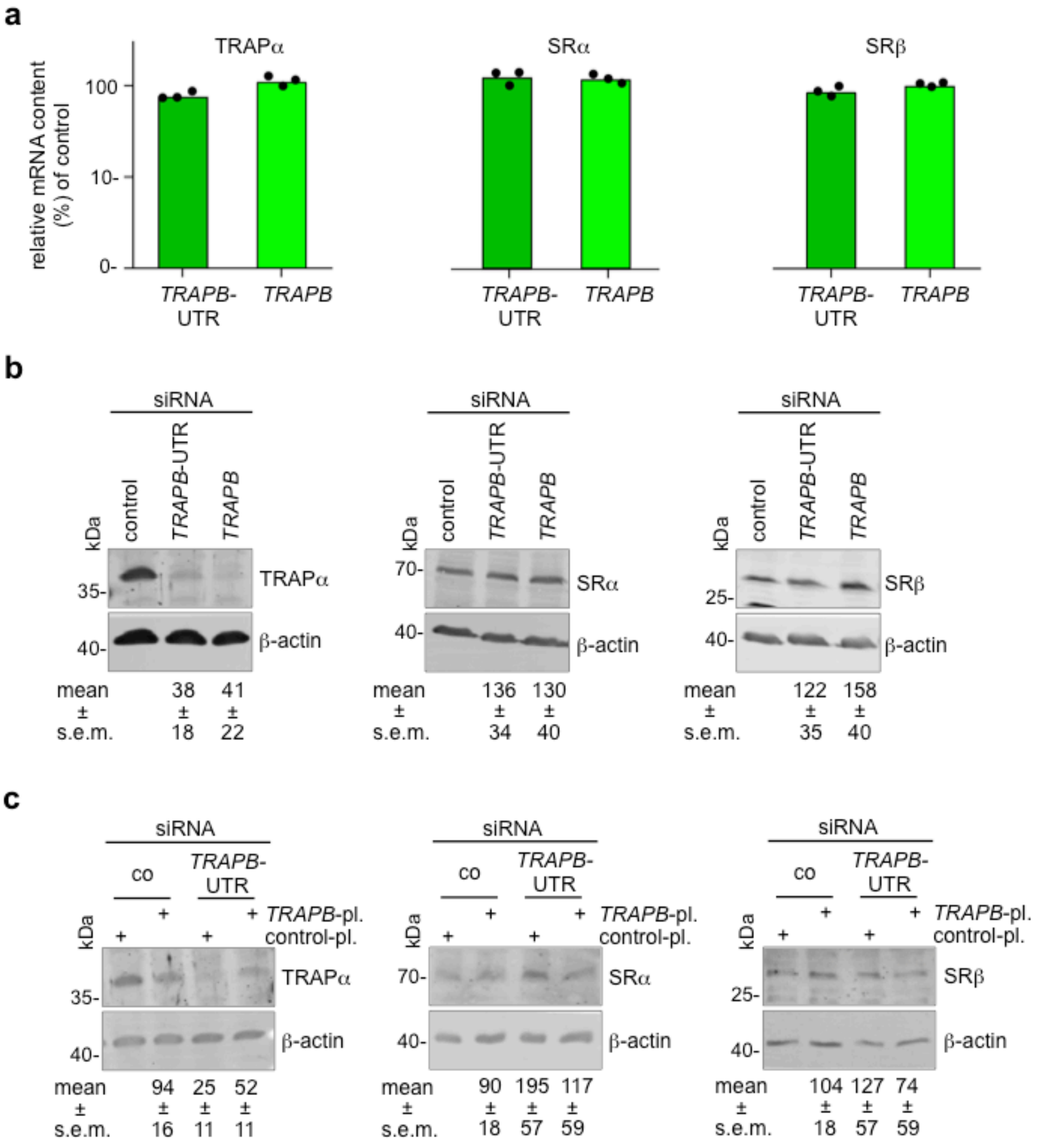

Supplementary Figure 3 | Validation of mass spectrometry data by western blot and quantitative RT-PCR. The color coding follows Fig. 1. (**a**–**c**) HeLa cells were depleted of TRAP-complex using two different *TRAPB*-targeting siRNAs in parallel to treatment with a

non-targeting siRNA, and the consequences were analyzed by quantitative RT-PCR and western blots for TRAP and SR subunits. (**a**) Quantitative RT-PCR data represent the mean mRNA values relative to control and the corresponding dot plots for a total of nine replicates for each siRNA from three independent experiments. (**b, c**) Quantitative western blot data represent the mean protein levels relative to control and standard errors of the mean (s.e.m.) for >7 (**b**) or 3 (**c**) independent experiments. (**c**) Silencing phenotypes were rescued by the indicated complementation and analyzed by western blot.

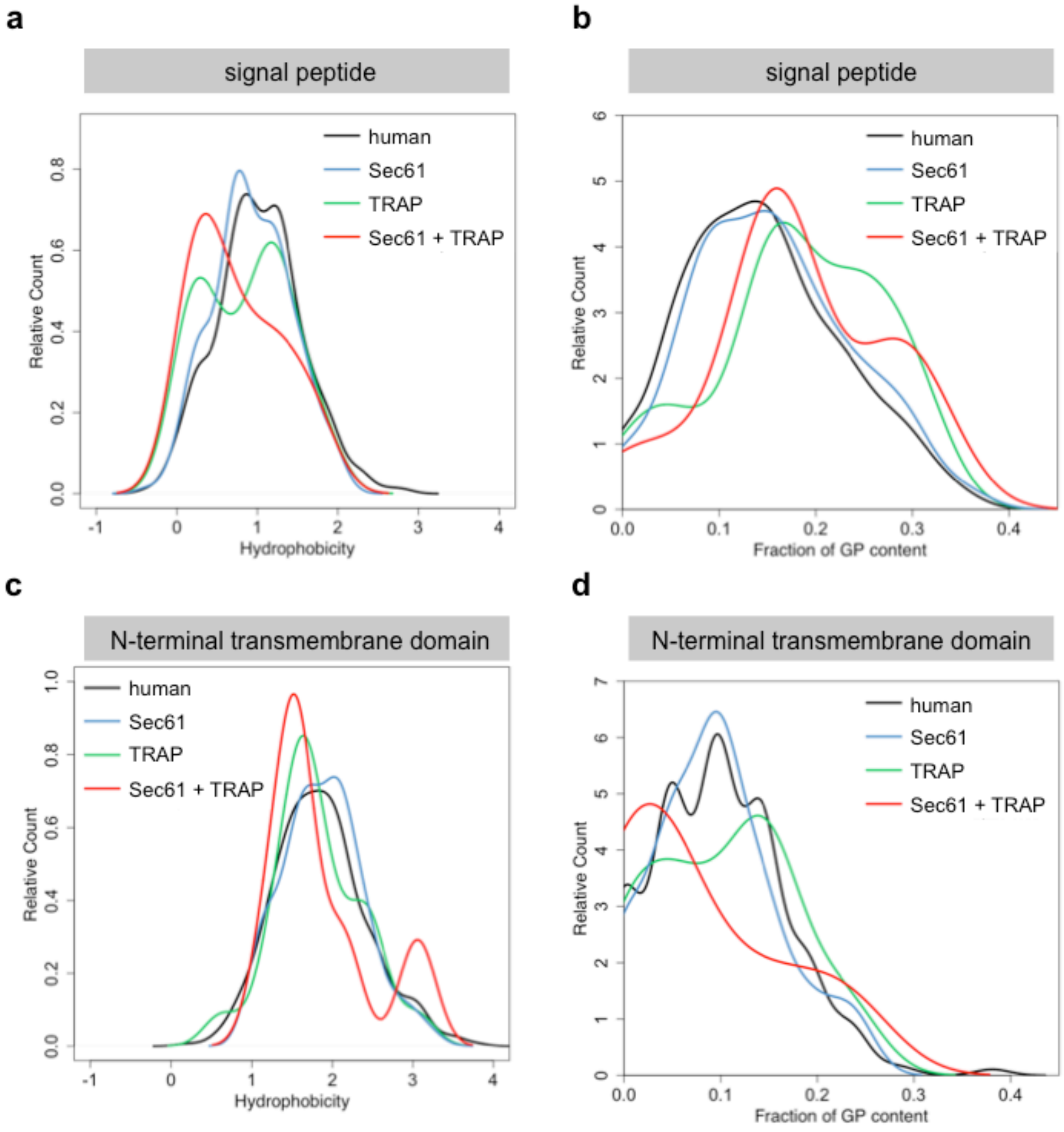

Supplementary Figure 4 | Physicochemical properties of Sec61 and TRAP clients with SP or TMH. The color coding follows Fig. 1. Using custom scripts, we computed the hydrophobicity score (**a, c**) and glycine/proline (GP) content (**b, d**) of the sequences of signal

peptides (**a, b**) or transmembrane helices (TMH) (**c, d**). The hydrophobicity score of a peptide was calculated as the averaged hydrophobicity of its amino acids according to the well-known Kyte-Doolittle propensity scale. GP content was calculated as the total fraction of glycine and proline in the respective sequence.

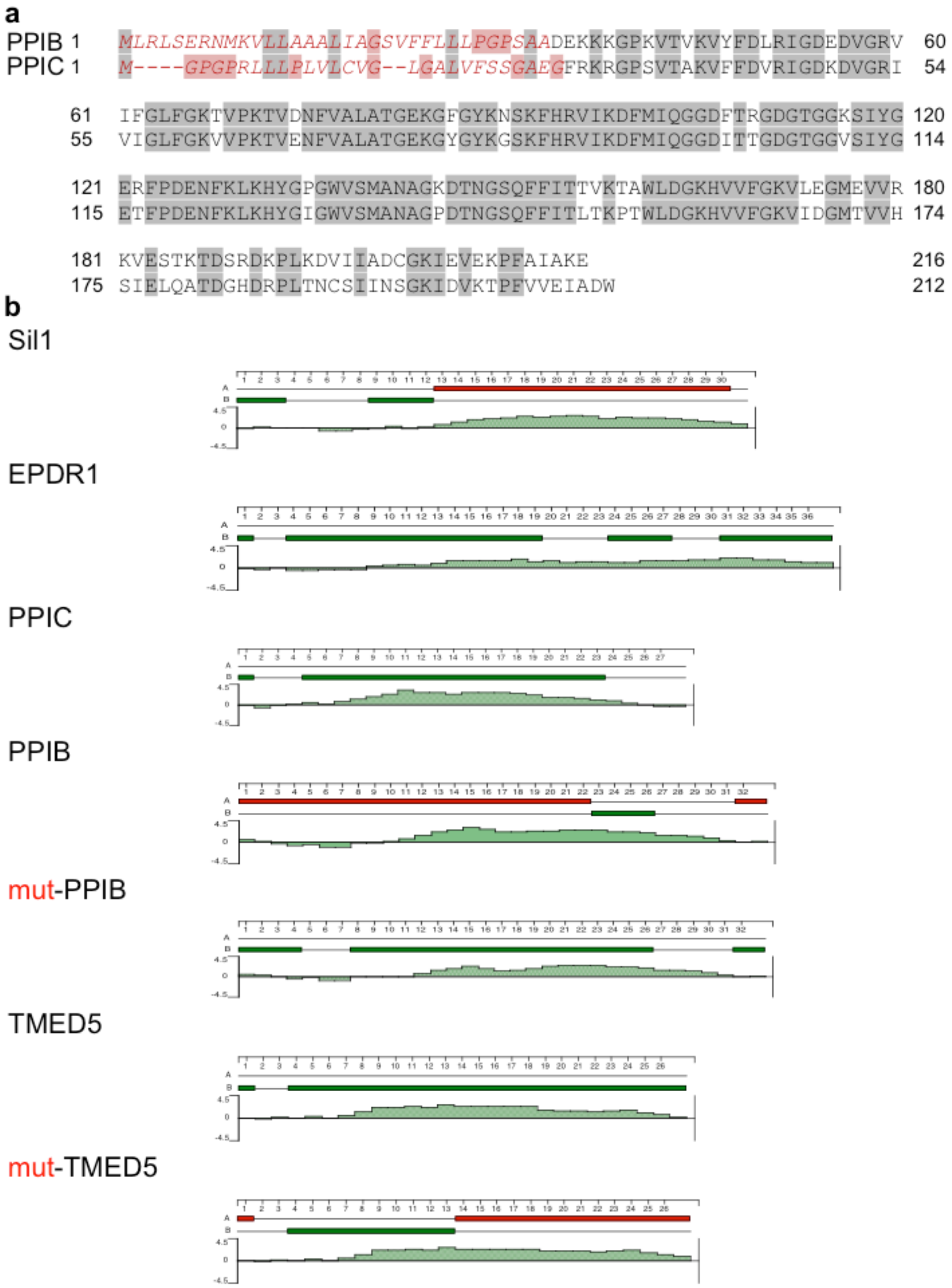

Supplementary Figure 5 | Properties of TRAP clients. (a) The amino acid sequence alignment of PPIB and PPIC was carried out using Clustal V of the DNASTAR software (Lasergene 12). The signal peptides are shown in red italics, glycine and proline residues in

the SP are highlighted in red, identical amino acids are highlighted in grey. (**b**) The propensities for helix and ß sheet formation as well as the hydrophobicities of SP of the indicated model precursor polypeptides were determined with the protean prediction tool of the DNASTAR software (Lasergene 12) following Garnier *et al*. (1978)[1] and Kyte & Doolittle (1982)[2], respectively. The number of amino acid residues are shown in the first line, predicted alpha helical regions in red in the second line, predicted beta sheets in the third line and hydrophobicity plots in the last. The lengths of SP are in scale.

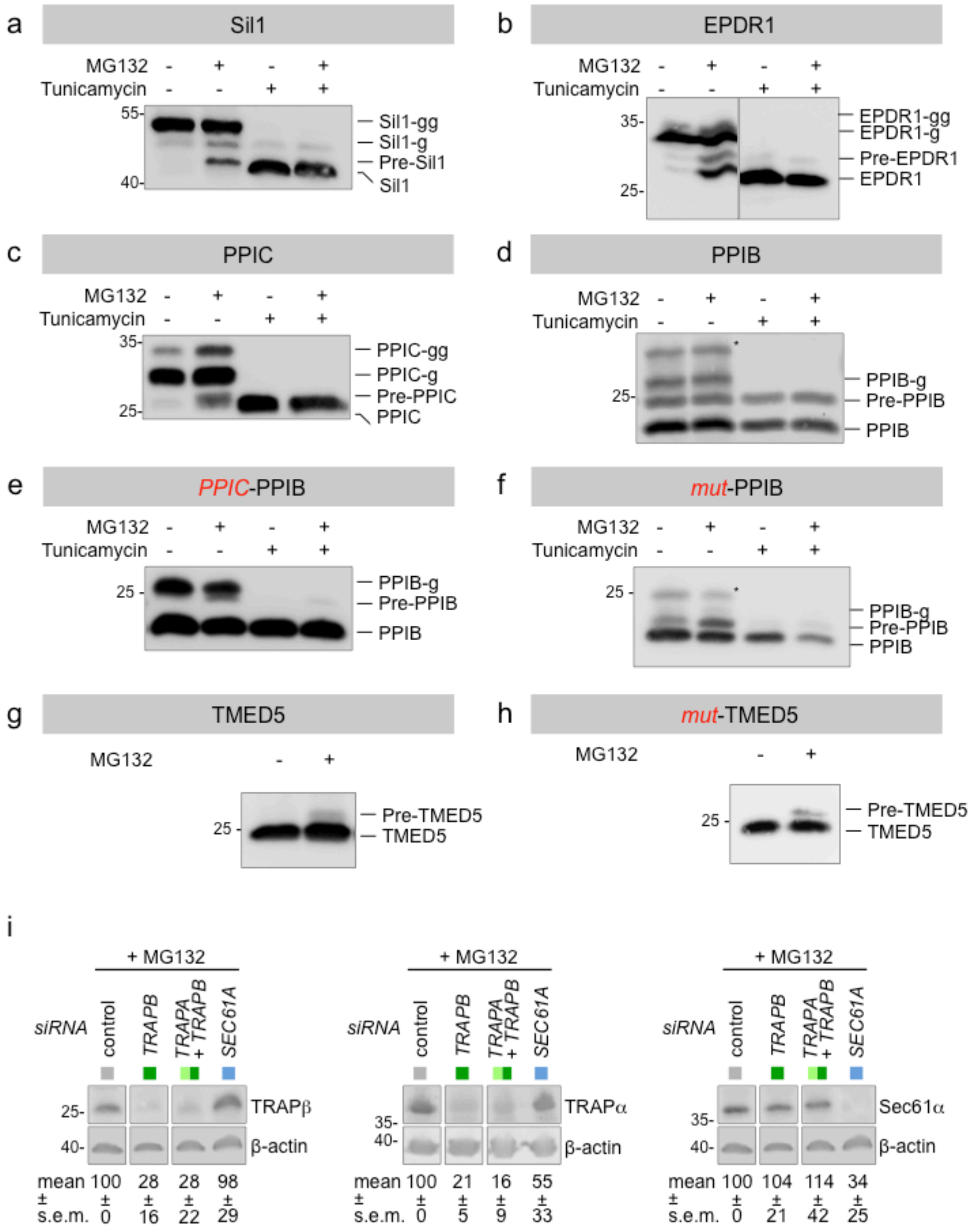

Supplementary Figure 6 | Identification of precursors of TRAP clients and their mature forms. (**a-h**) Plasmid driven over-production of model precursor polypeptides in HeLa cells was carried out in the presence of the indicated siRNA for 24 h. Where indicated, MG 132

and Tunicamycin were present during the last 8 and 23 h, respectively. Samples were analyzed by western blot. We note that the pre-form represents the precursor polypeptide, the form without any addition the mature protein without N-gylcosylation, g a mono-gylcosylated mature protein, and gg a doubly-glycosylated mature protein. (**i**) Plasmid driven over-production of model precursor polypeptides in siRNA treated HeLa cells was carried out in the presence of the indicated siRNA for 24 h and MG 132 as described in the legend to Fig. 6. Samples were analyzed for depletion efficiency by western blot. Quantitative data represent the mean protein levels relative to control and standard errors of the mean (s.e.m.) for 4 independent experiments. *, unspecific band.

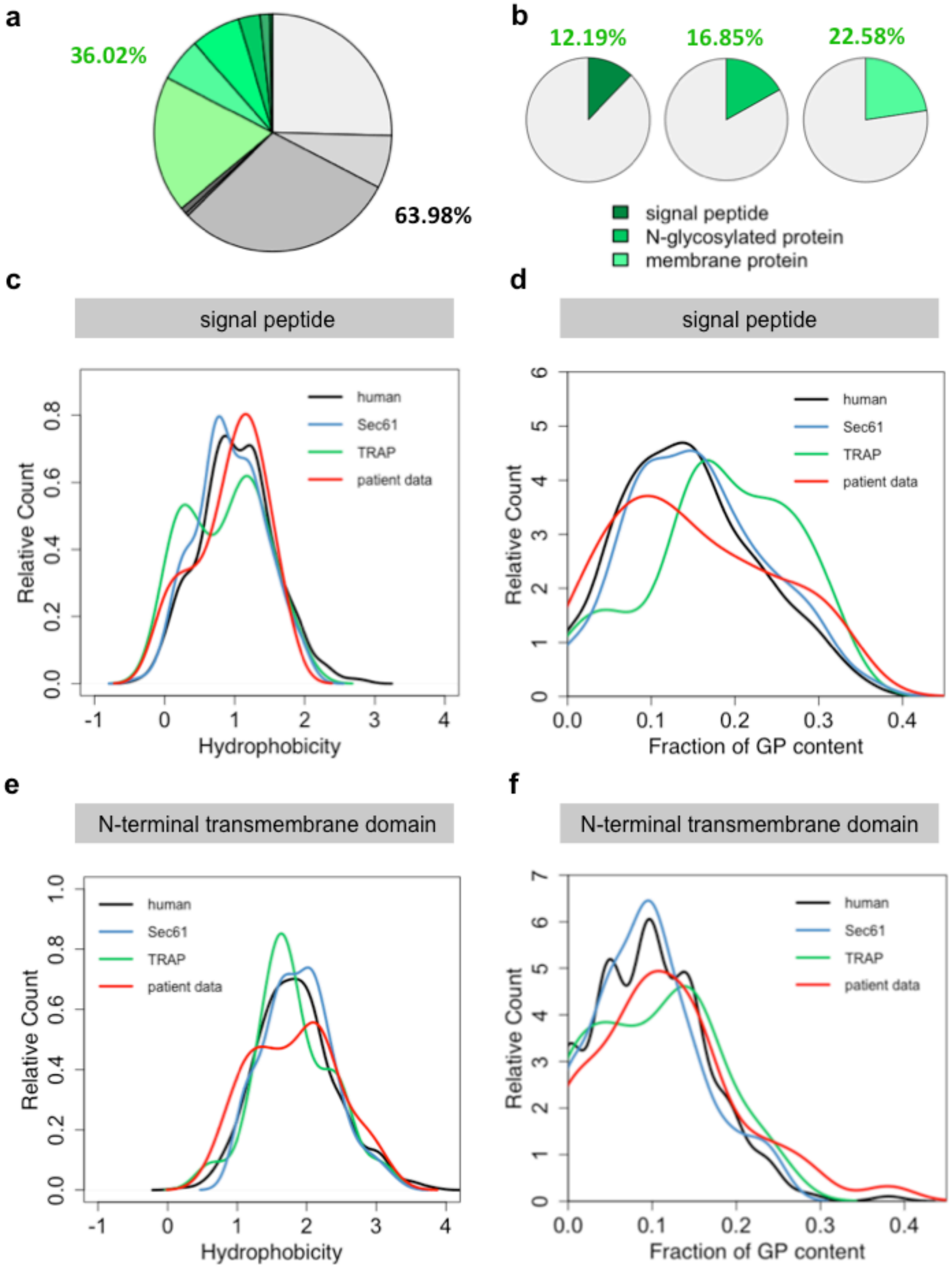

Supplementary Figure 7 | Validation of TRAP clients in CDG patient fibroblasts. (**a** and **b**) The color coding follows Fig. 1. Two control fibroblasts and three CDG patient fibroblasts with TRAP-deficiency were subjected to label-free quantitative proteomic analysis and

differential protein abundance analysis using the same analysis workflow as in Figs. 1 and 5. Protein annotations of signal peptides, membrane location, and N-glycosylation in humans were extracted from UniProtKB, and used to determine the enrichment of Gene Ontology annotations among the secondarily affected proteins. Using custom scripts, we computed the hydrophobicity score (**c, e**) and glycine/proline (GP) content (**d, f**) of the sequences of signal peptides (**c, d**) or transmembrane helices (TMH) (**e, f**). The hydrophobicity score of a peptide was calculated as the averaged hydrophobicity of its amino acids according to the well-known Kyte-Doolittle propensity scale. GP content was calculated as the total fraction of glycine and proline in the respective sequence.

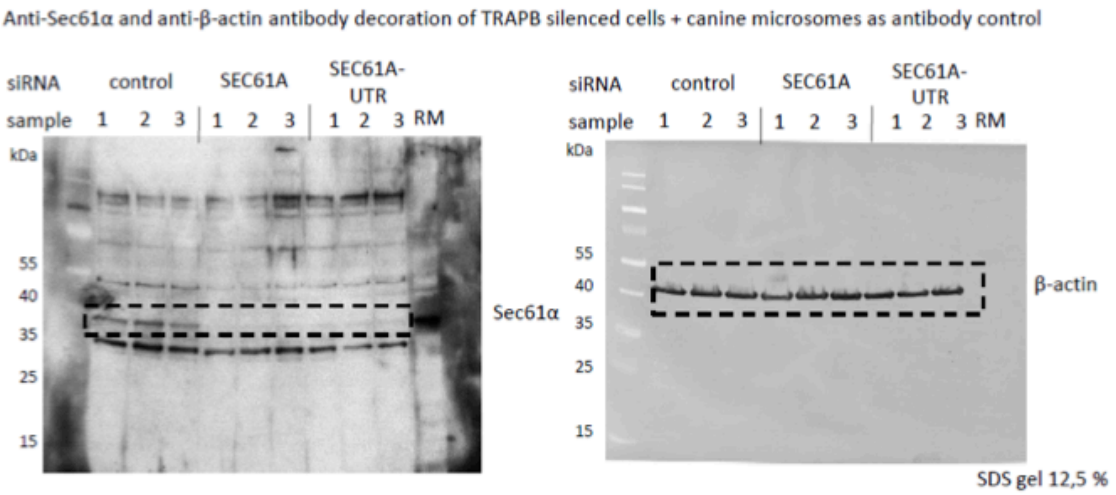

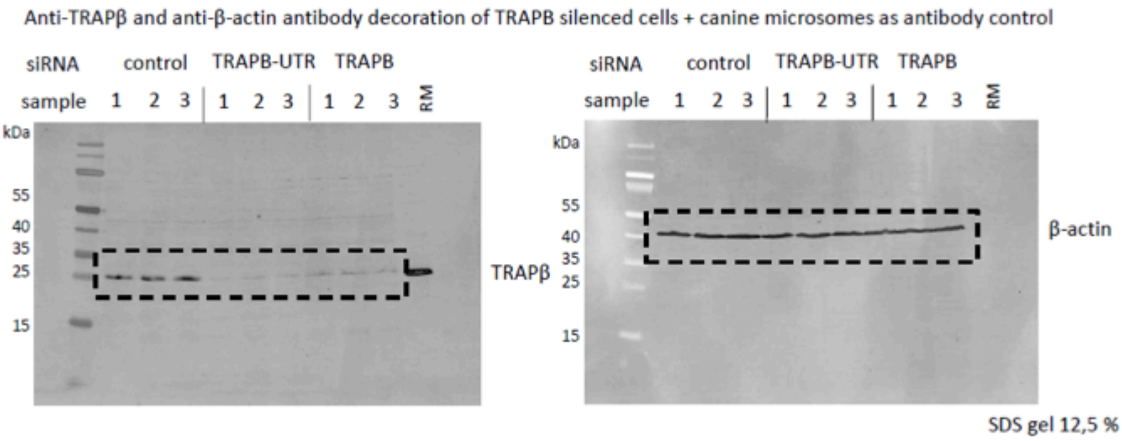

Supplementary Figure 8 | Full scans for Western blots which are shown in Fig. 1c, e.

Cropped areas are indicated.

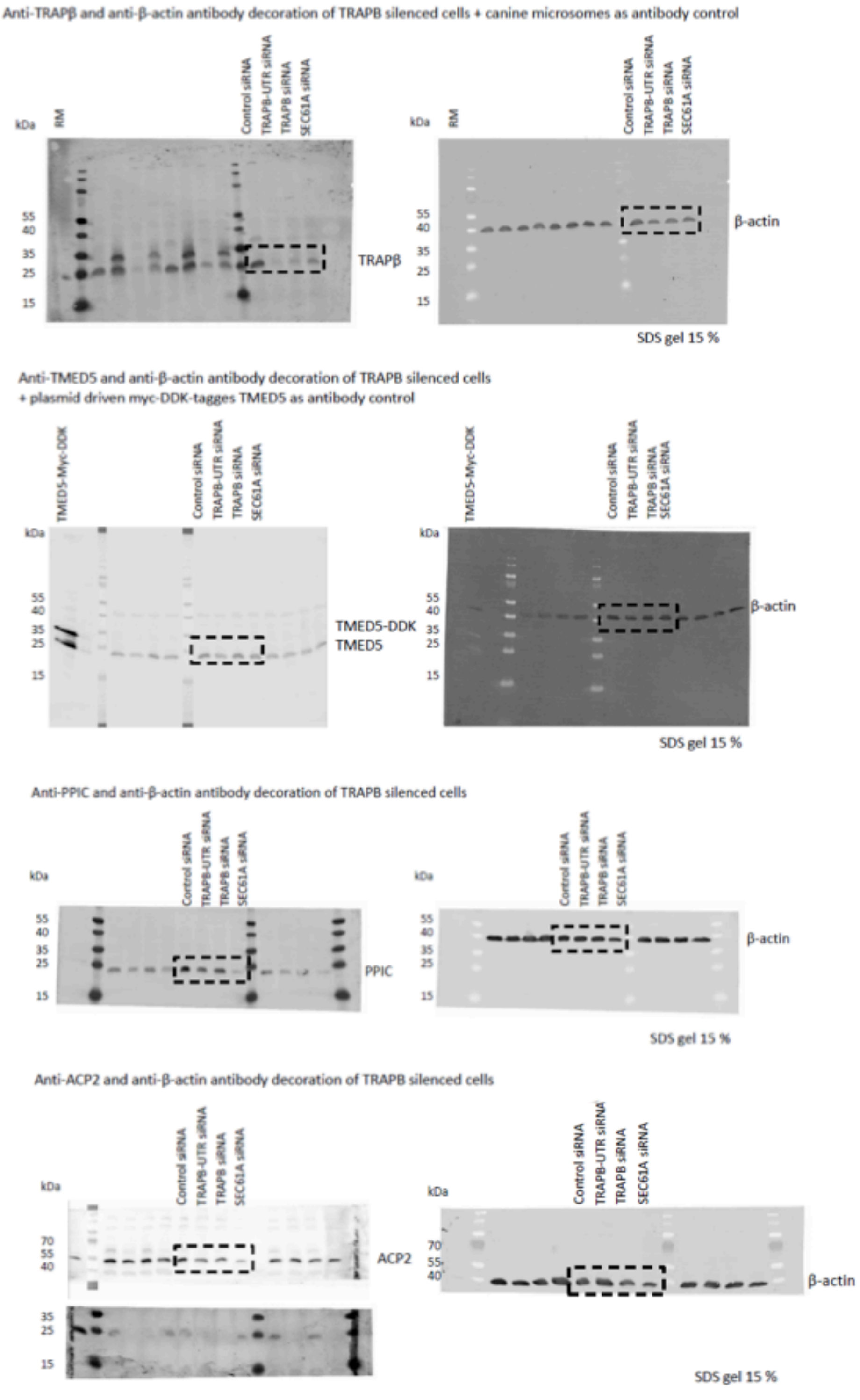

Supplementary Figure 9 | Full scans for Western blots which are shown in Fig. 3b.

Cropped areas are indicated.

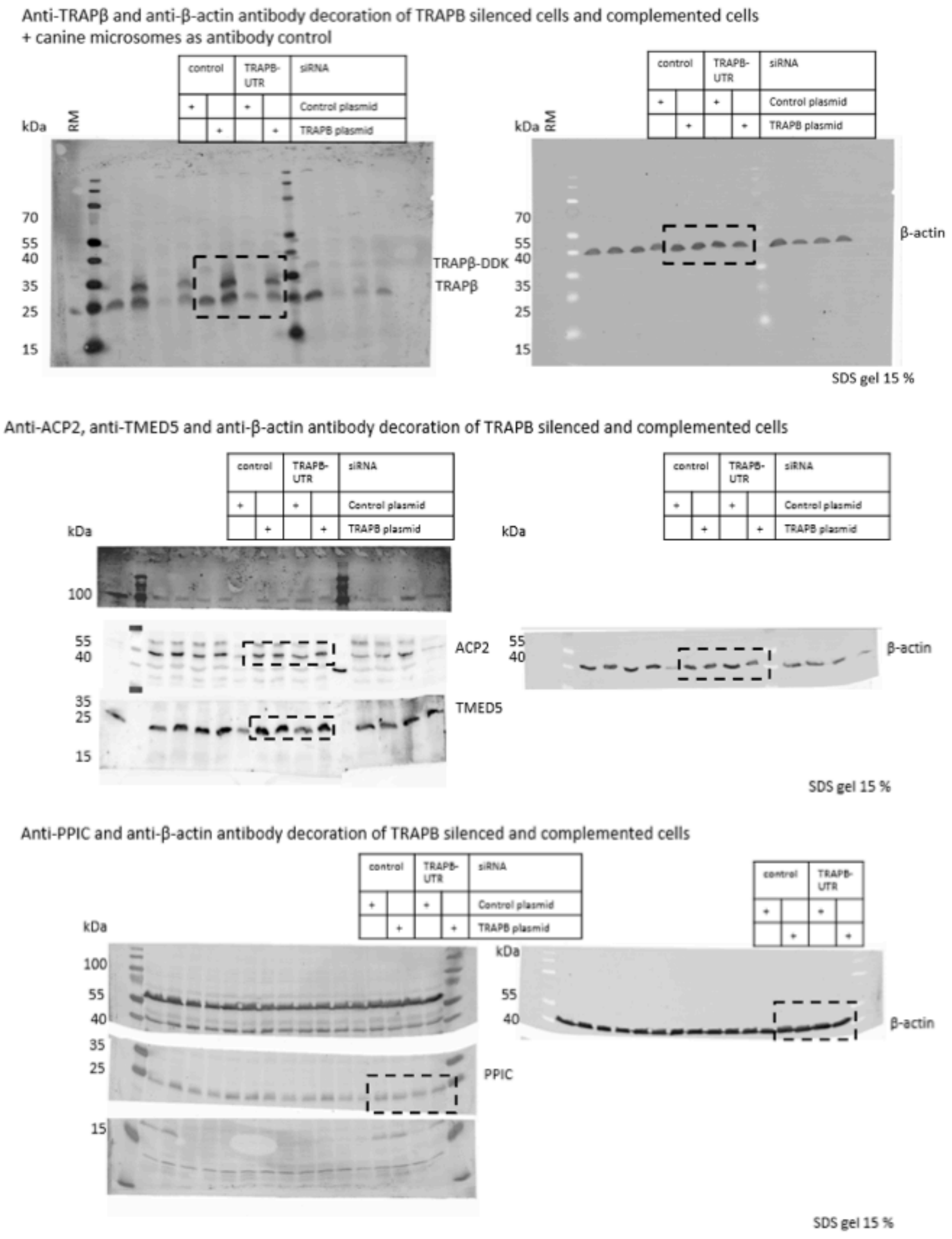

Supplementary Figure 10 | Full scans for Western blots which are shown in Fig. 3c.

Cropped areas are indicated.

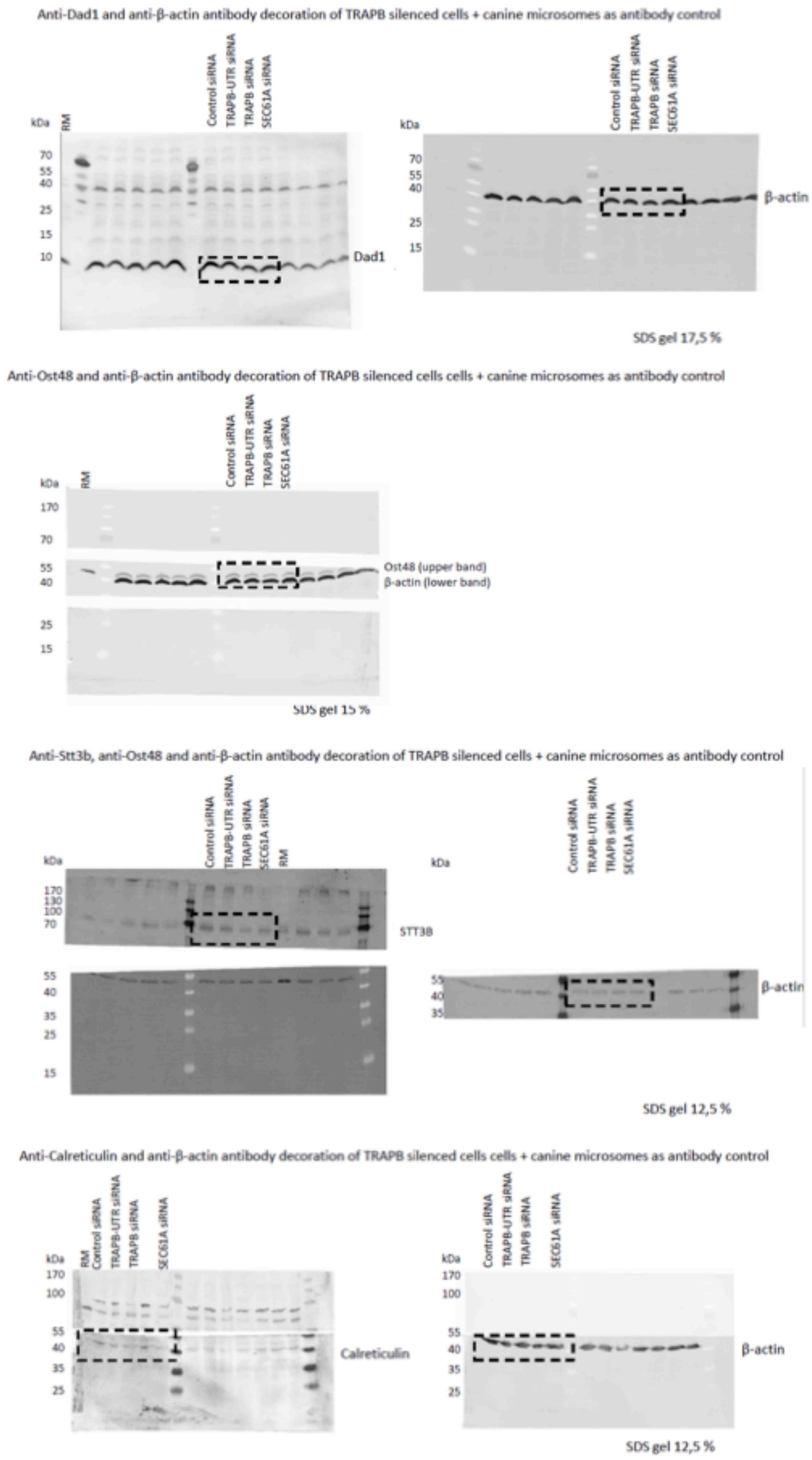

Supplementary Figure 11 | Full scans for Western blots which are shown in Fig. 4a.

Cropped areas are indicated.

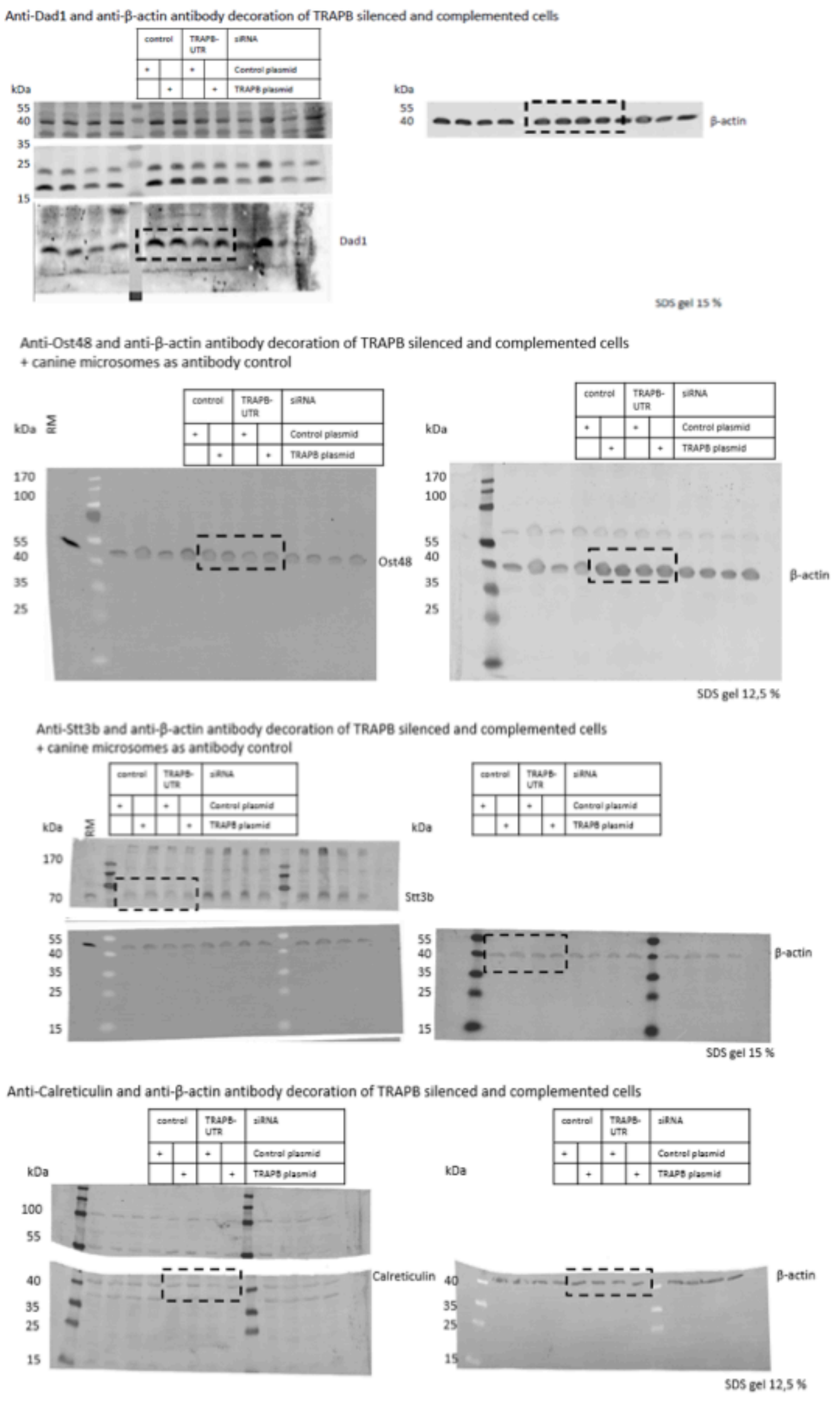

Supplementary Figure 12 | Full scans for Western blots which are shown in Fig. 4b.

Cropped areas are indicated.

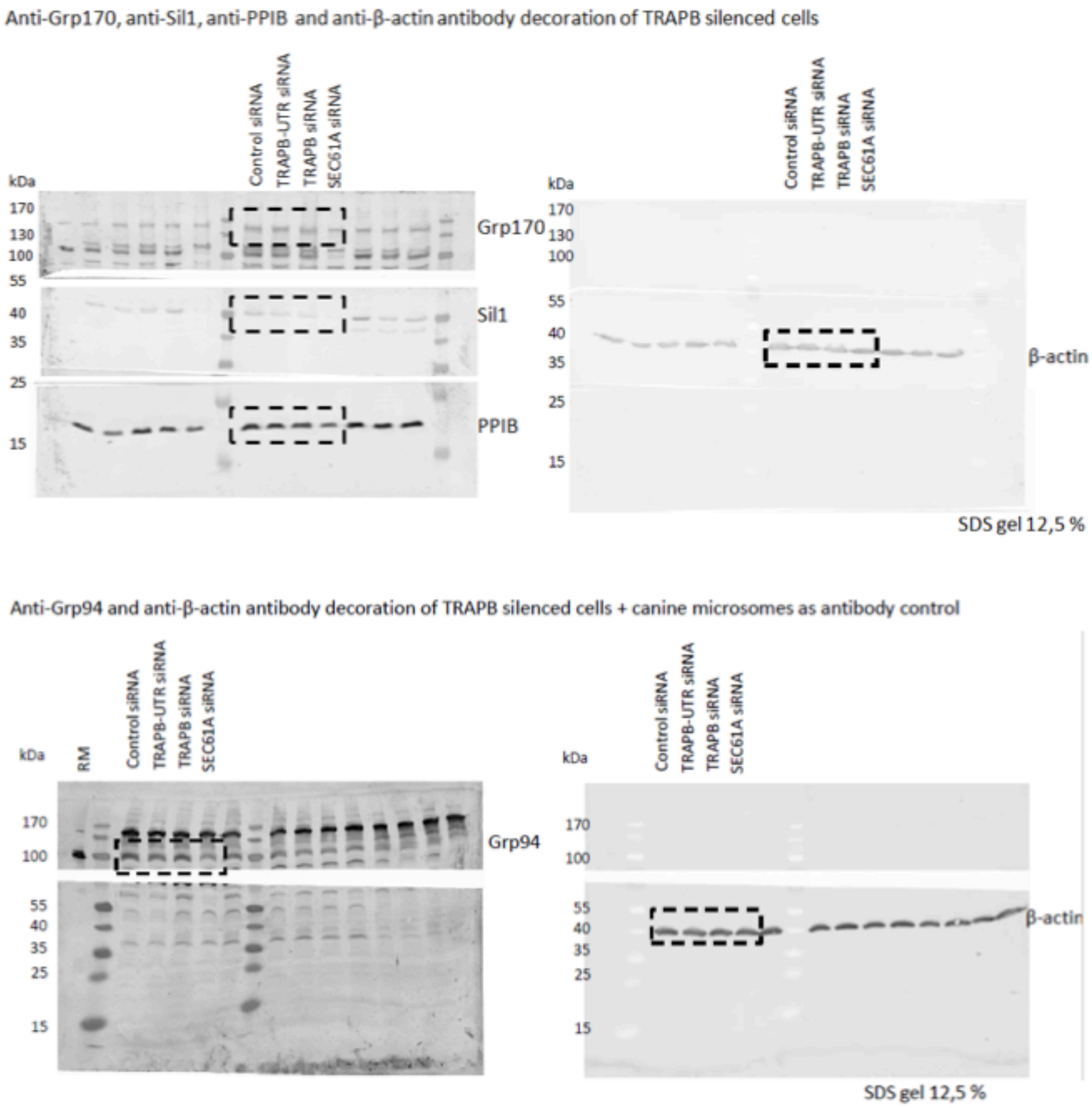

Supplementary Figure 13 | Full scans for Western blots which are shown in Fig. 4d.

Cropped areas are indicated.

Anti-Sil1 and anti-β-actin antibody decoration of TRAPB silenced and complemented cells

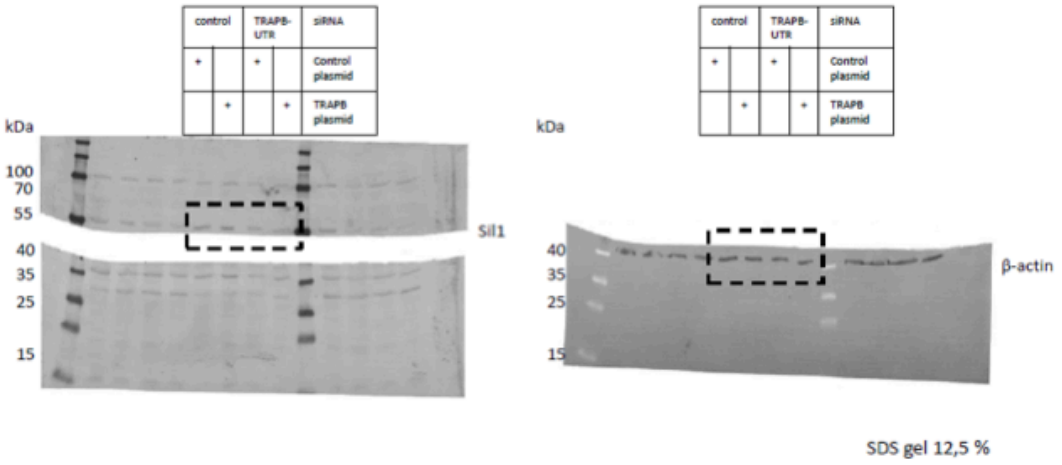

Supplementary Figure 14 | Full scans for Western blots which are shown in Fig. 4e.

Cropped areas are indicated.

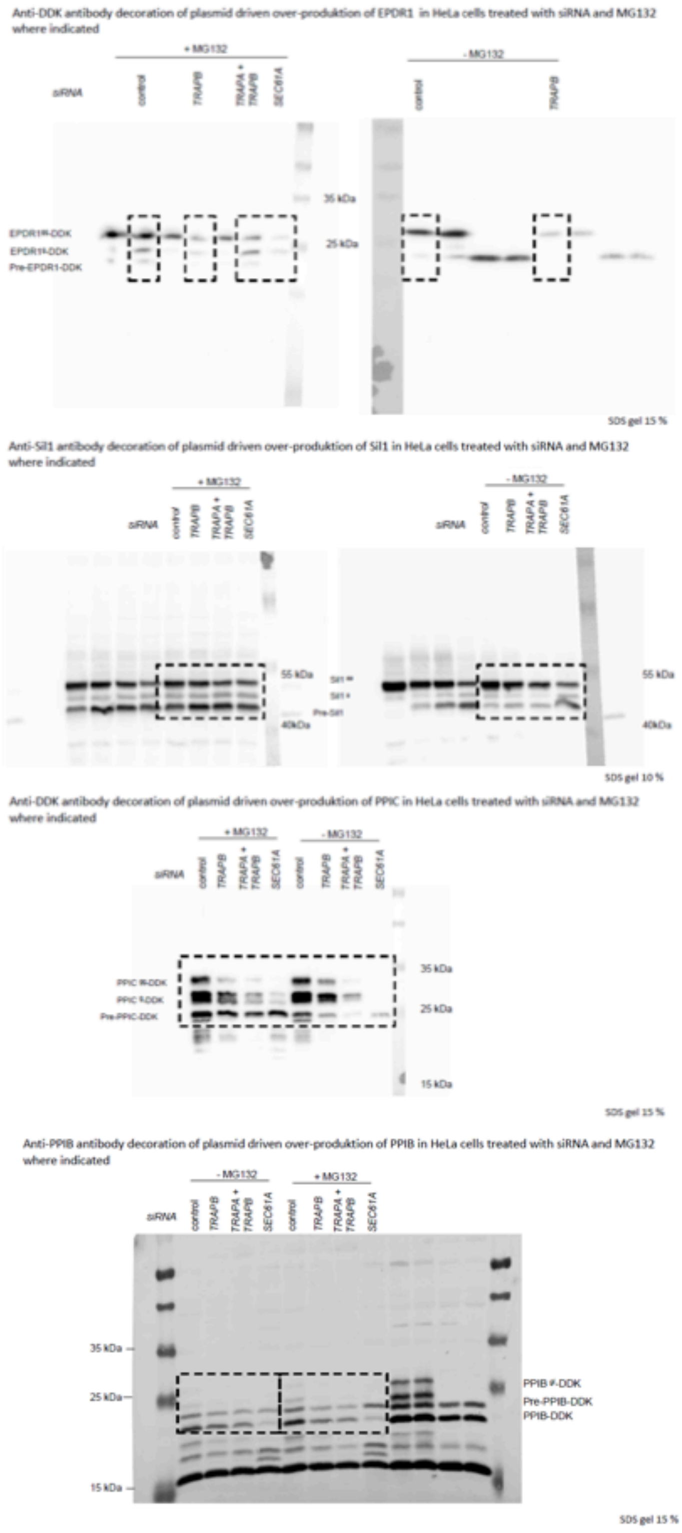

Supplementary Figure 15 | Full scans for Western blots which are shown in Fig. 6a-d.

Cropped areas are indicated.

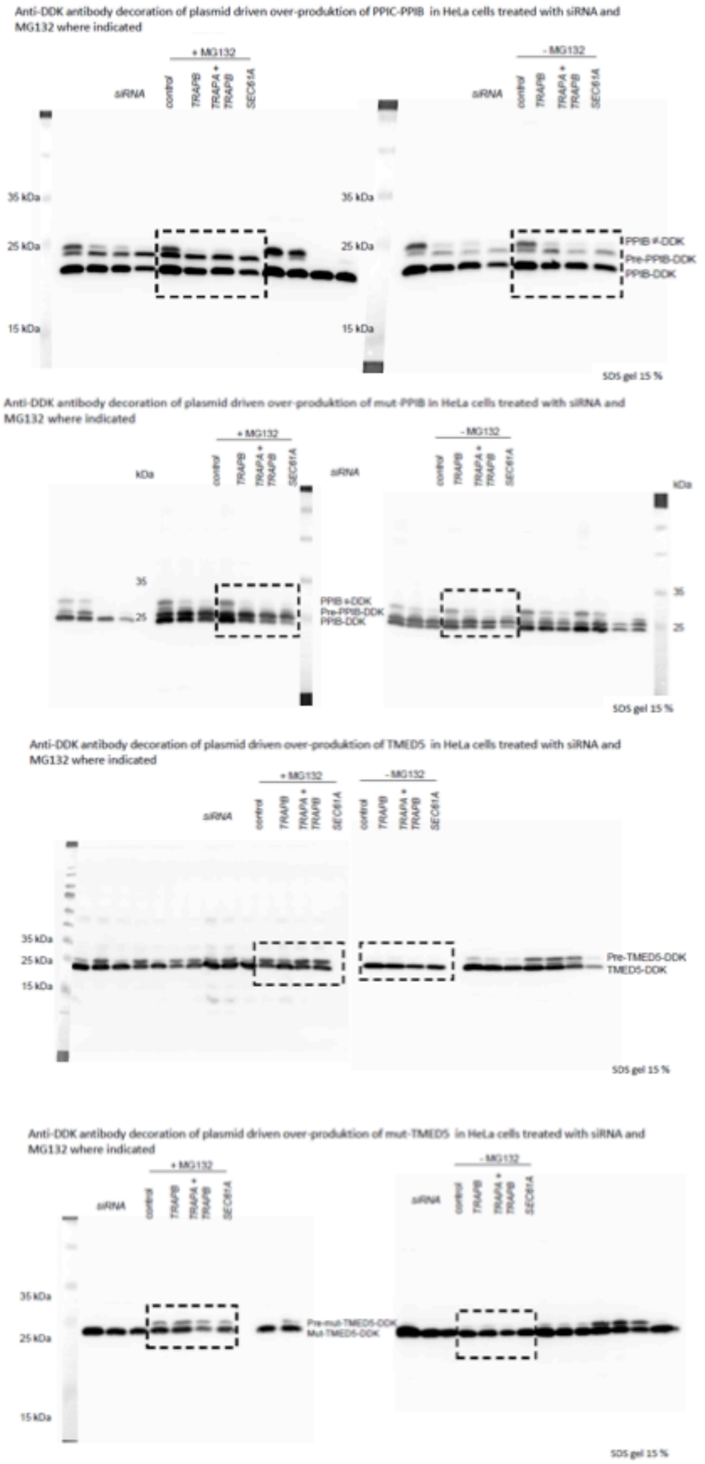

Supplementary Figure 16 | Full scans for Western blots which are shown in Fig. 6e-h.

Cropped areas are indicated.

Anti-TRAPß, anti-Sec61α and anti-ß-actin antibody decoration in TRAPB and SEC61A1 silenced cells

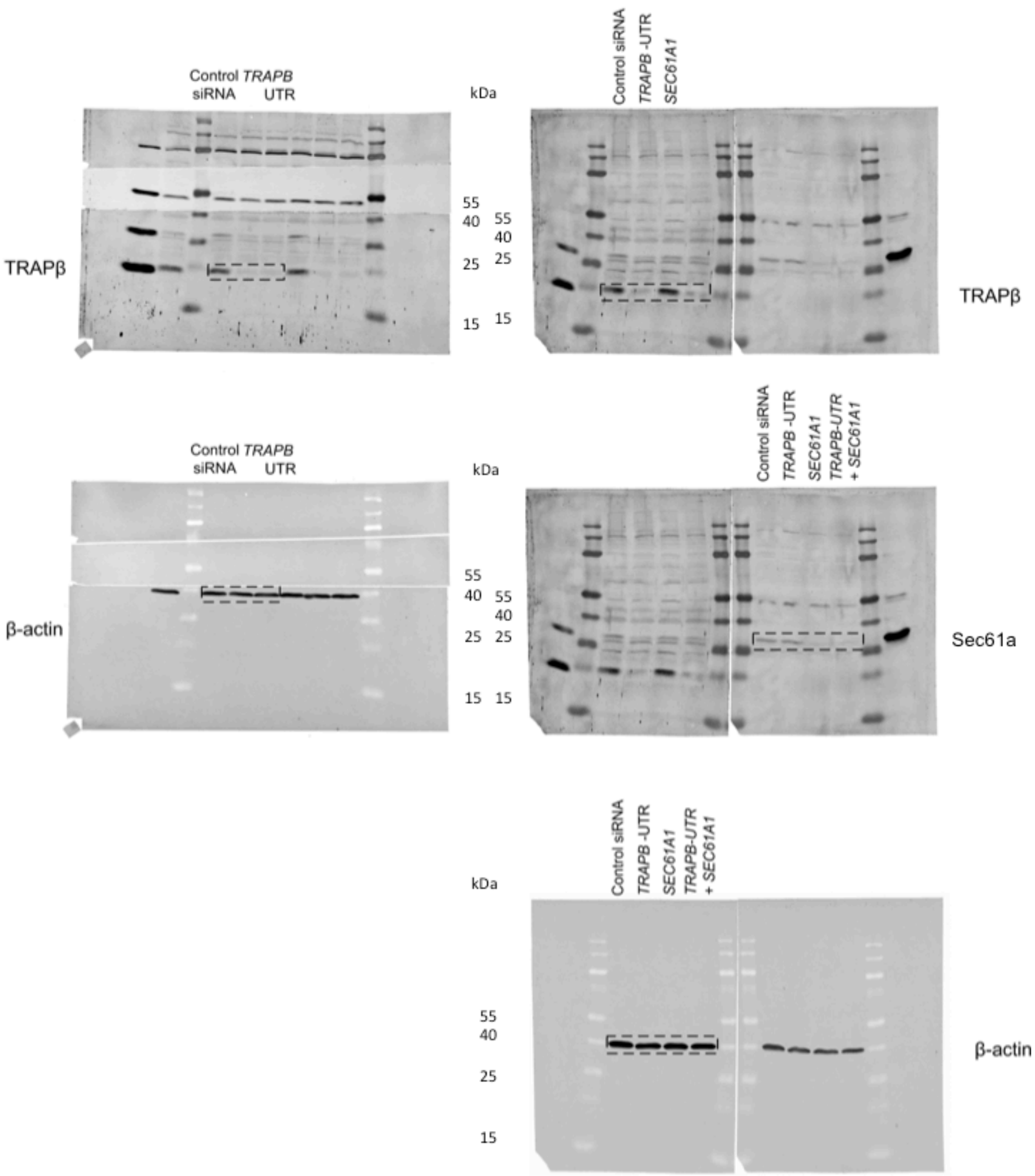

Supplementary Figure 17 | Full scans for Western blots which are shown in Fig. 7e, f.

Cropped areas are indicated.

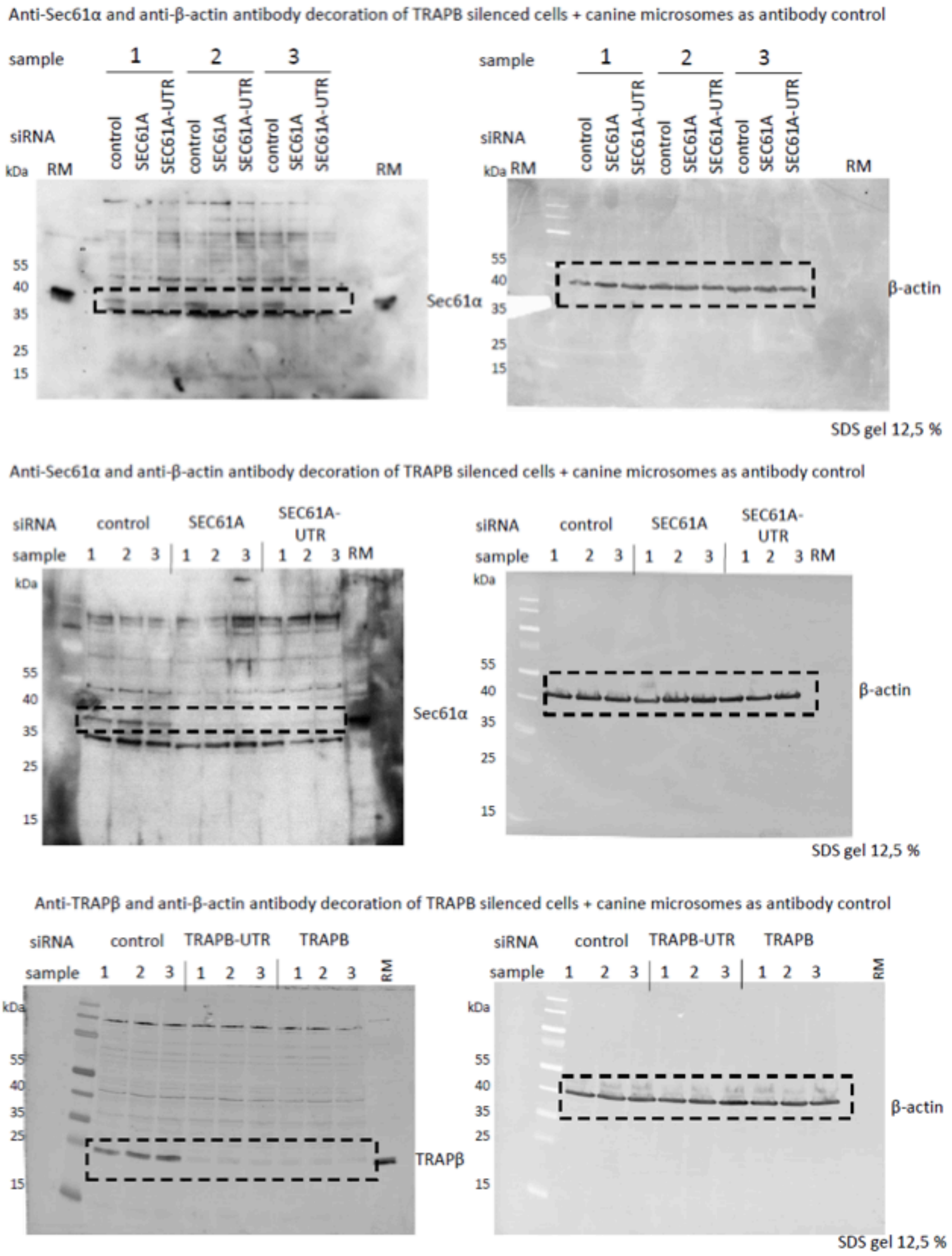

Supplementary Figure 18 | Full scans for Western blots which are shown in Supplementary Fig. 1a, d. Cropped areas are indicated.

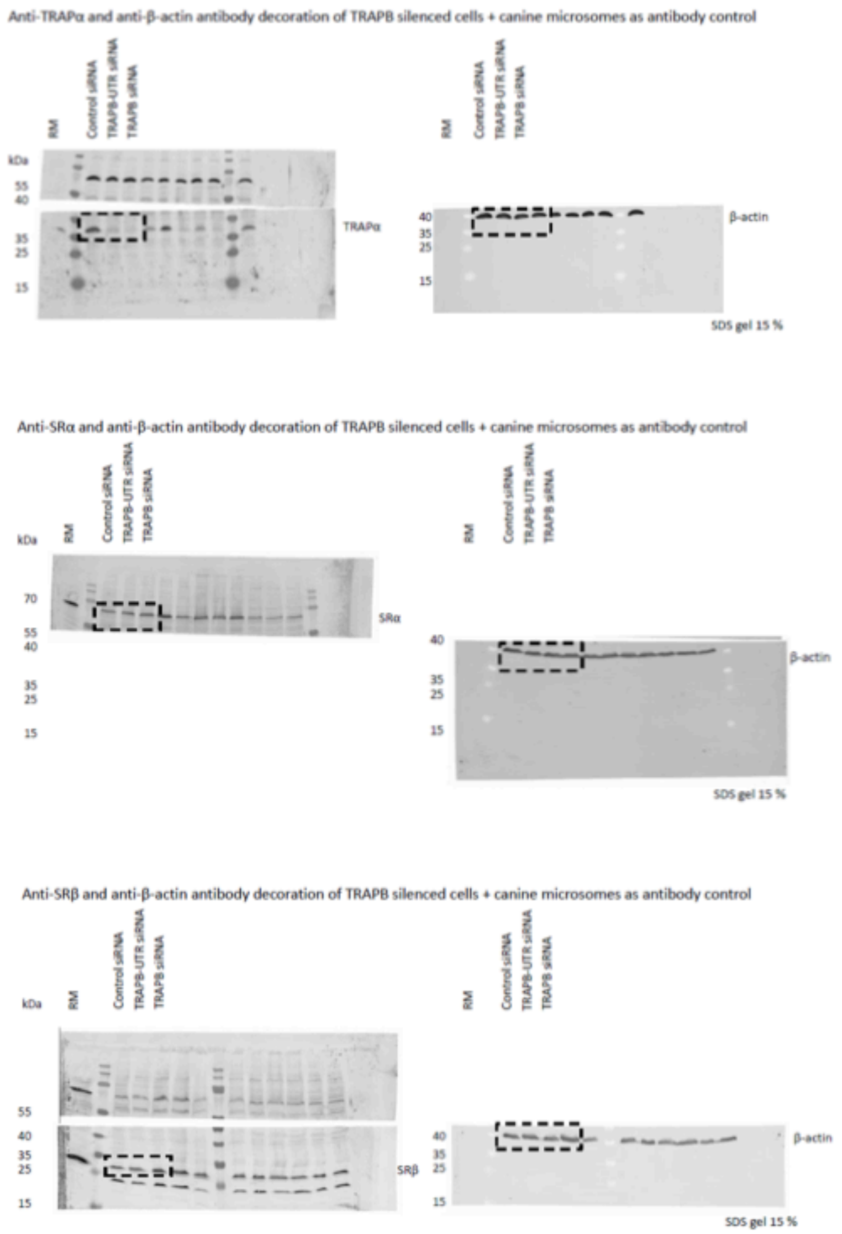

Supplementary Figure 19 | Full scans for Western blots which are shown in Supplementary Fig. 3b. Cropped areas are indicated.

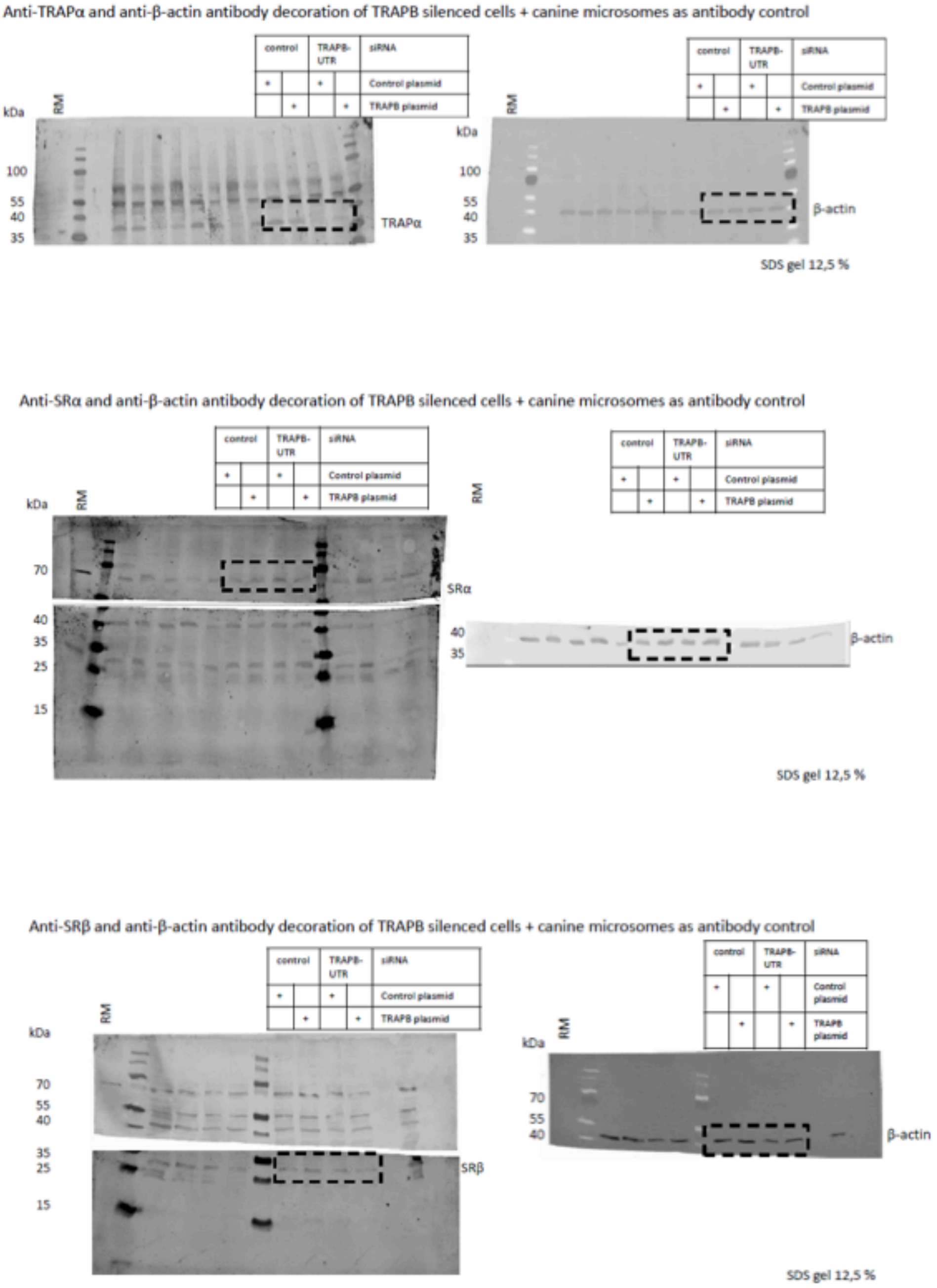

Supplementary Figure 20 | Full scans for Western blots which are shown in Supplementary Fig. 3c. Cropped areas are indicated.

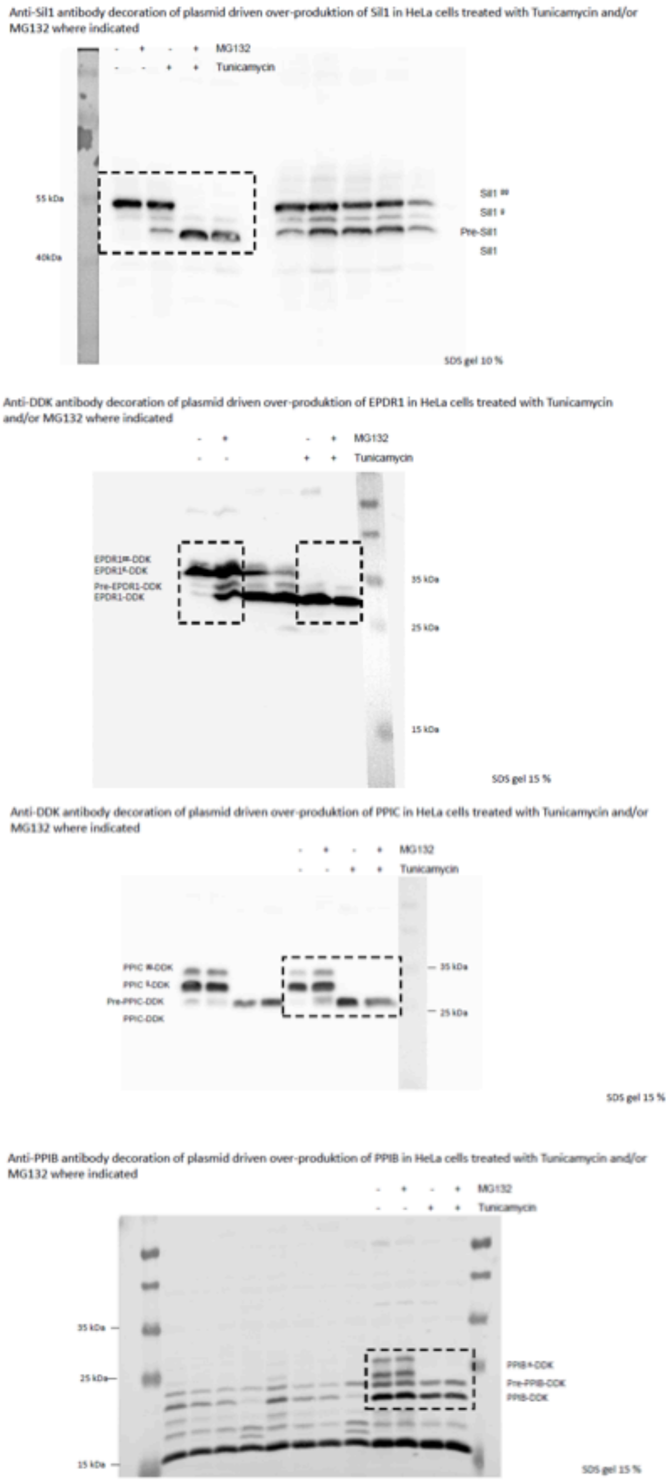

Supplementary Figure 21 | Full scans for Western blots which are shown in Supplementary Fig. 6a-d. Cropped areas are indicated.

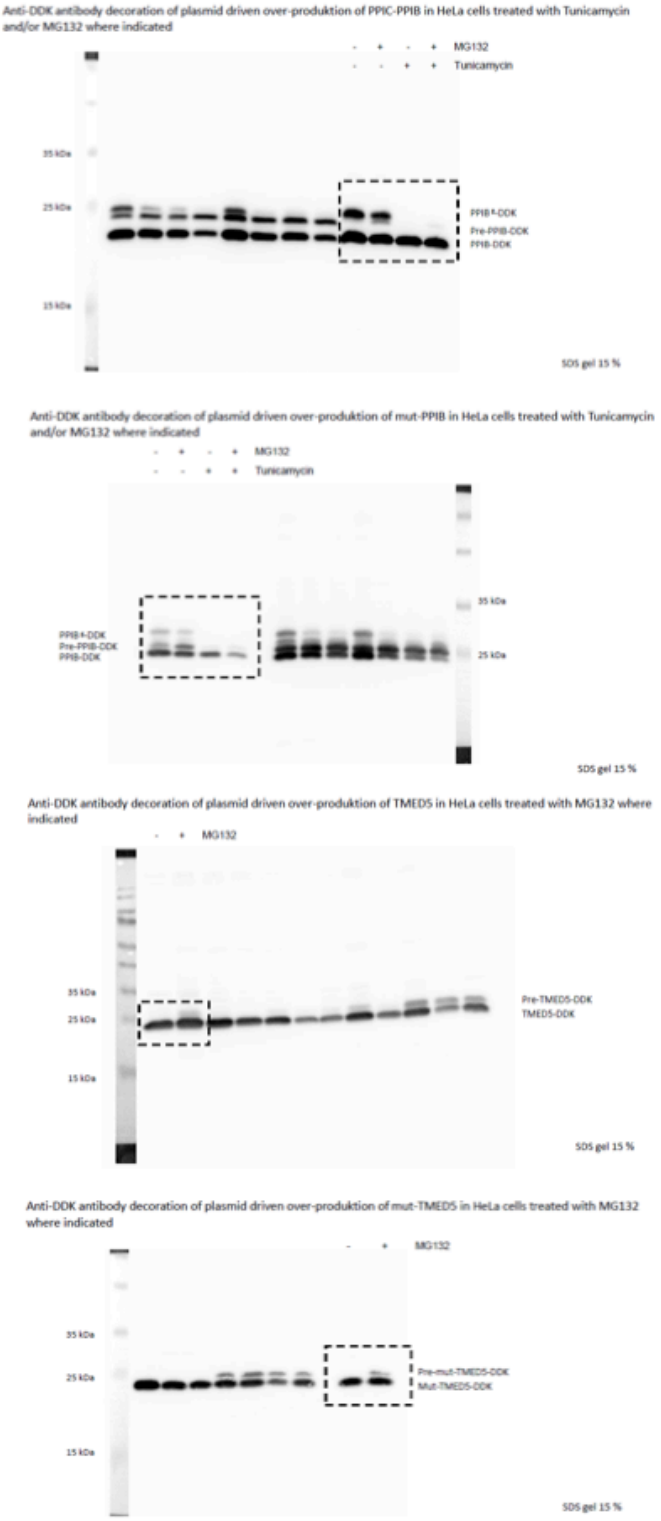

Supplementary Figure 22 | Full scans for Western blots which are shown in Supplementary Fig. 6e-h. Cropped areas are indicated.

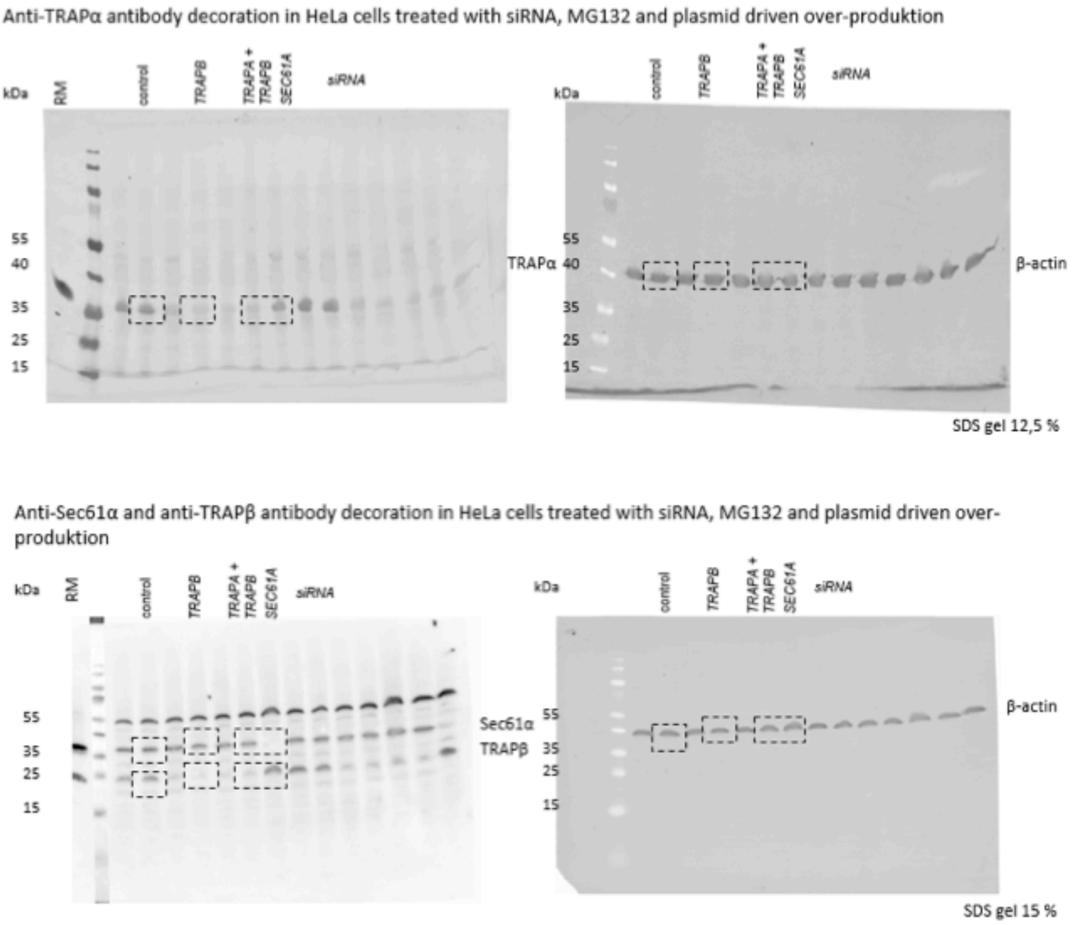

Supplementary Figure 23 | Full scans for Western blots which are shown in Supplementary Fig. 6i. Cropped areas are indicated.

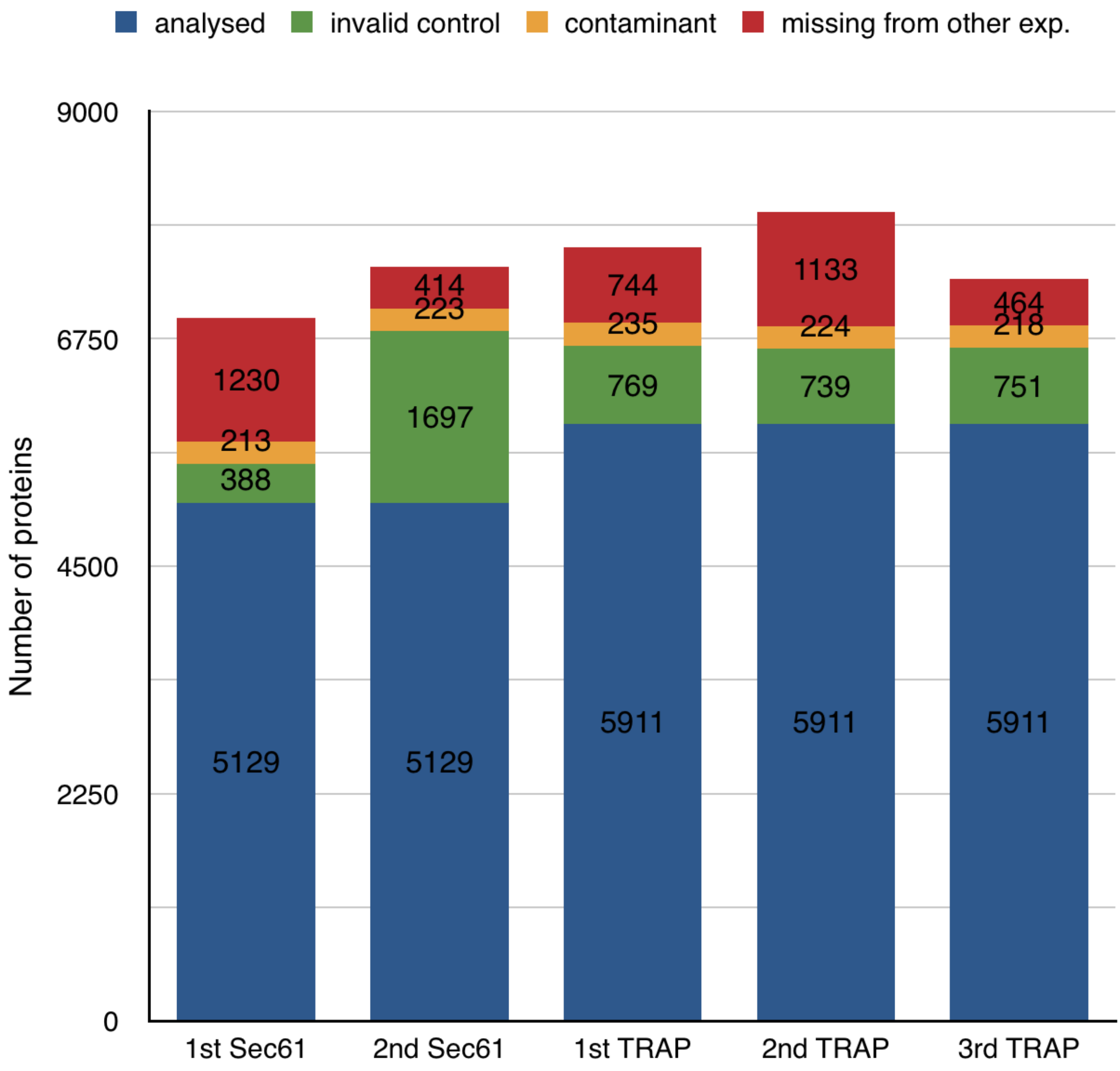

Supplementary Figure 24 | Number of proteins in the MS experiments. The Figure provides a detailed listing for the number of proteins detected in the two Sec61 depletion experiments (two leftmost columns) and in the three TRAP depletion experiments (three rightmost columns). The blue bars represent the proteins that we analyzed in this study. Marked in green are the proteins that do not have sufficient control data points, i.e. more than 2/3 of the control samples have missing data points. Marked in yellow are "contaminants" from MaxQuant analysis. Marked in red are proteins that cannot be found (or contain "invalid control") in other corresponding experiments. The number of proteins detected in Sec61 and

TRAP silencing experiments was 7212 ± 356 and 7670 ± 332, respectively (mean values with standard deviation, n=2 and n=3, respectively). The observed difference of about 460 was just a bit outside of the standard deviation and hence not statistically significant.

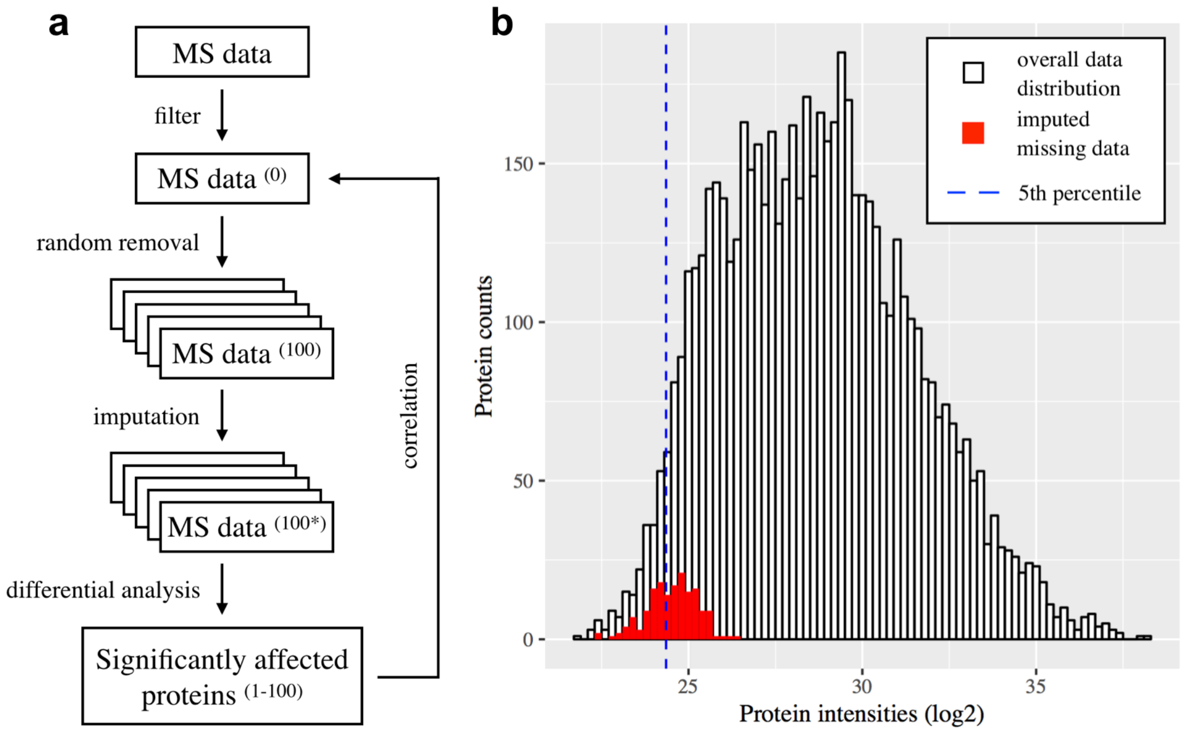

Supplementary Figure 25 | Validation of the used imputation method. (a) The imputation validation workflow. (**b**) The histograms denote the overall mass-spec data distribution (white) and the imputed data (red). The missing data were imputed at the lower tail of the overall distribution (at the 5th percentile in this case) based on the assumption that they come from proteins which have limited number of copies that cannot be detected by the mass spectrometry instrument.

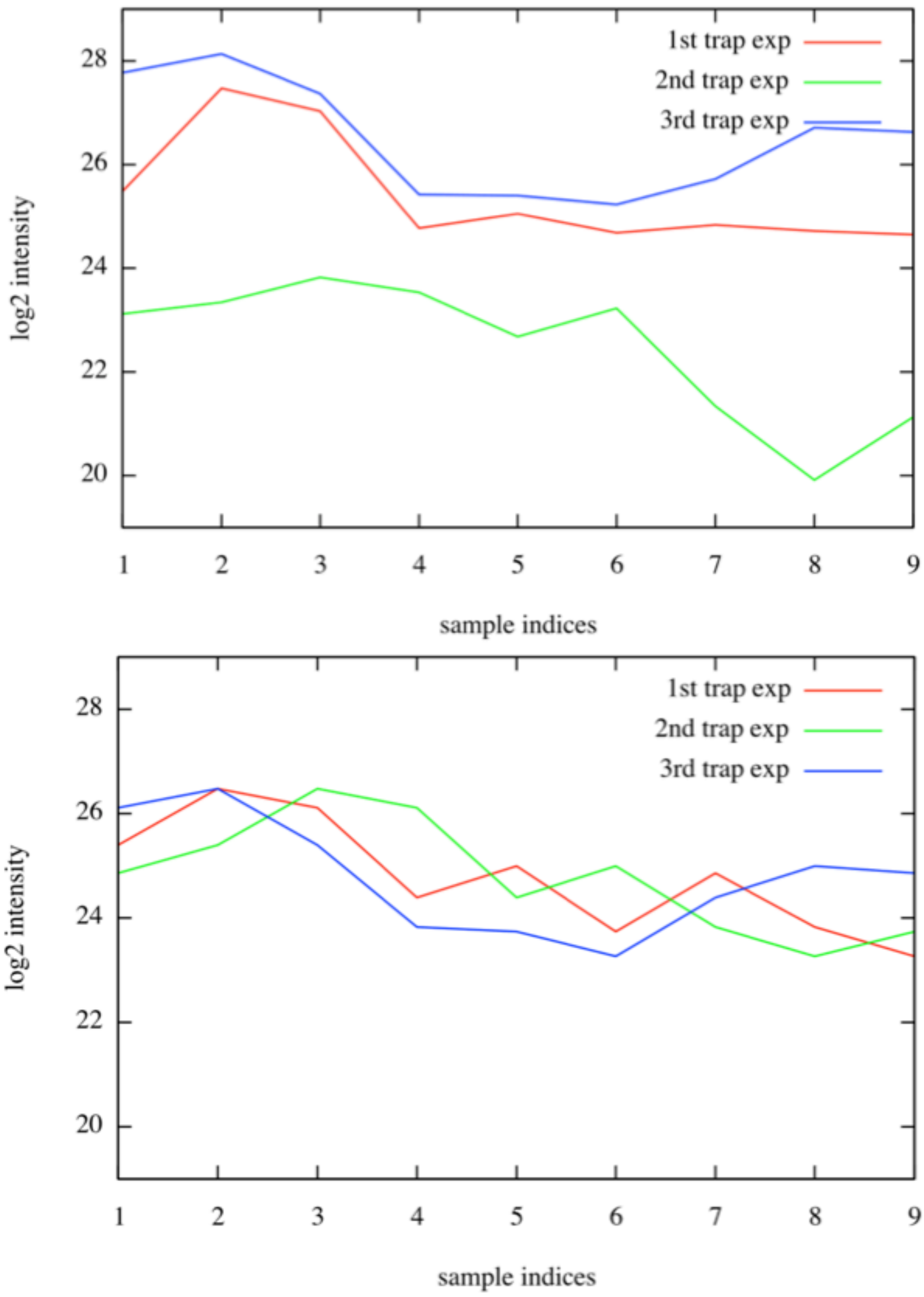

Supplementary Figure 26 | Protein-based quantile normalisation. The SSR2 intensity profile across all experiments (red - 1st experiment, green - 2nd experiment, blue - 3rd experiment) before and after quantile normalisation. The horizontal axis indicates sample

conditions: 1 to 3 - control, 4 to 6 – SSR2 silencing by 1st siRNA, 7 to 9 – SSR2 silencing by 2nd siRNA. The aim of the protein-based quantile normalisation is to remove the systematic variation among different experimental iterations (three iterations of TRAP silencing experiment). It ranks the raw data, computes the averages, and replaces the original values by the ranked averages. As a result, the distributions of one protein become statistically identical across all iterations. (**a**) contains raw MS data points for the SSR2 protein. (**b**) contains normalized data points for SSR2. The variation left after normalisation reflects the biological variation between samples. In (**a**), SSR2 levels of the controls (indices 1-3) are higher than both siRNAs in experiment 2 (red) and higher than the first siRNA in experiment 1 (blue). In the third experiment (green), the second siRNA (indices 7-9) induces lower levels than in the controls and the first siRNA. The same conclusions can be drawn from (**b**). The benefit of the normalized values in (**b**) is that the blue, red, and green distributions contain identical values. Thus, one can now apply standard statistical tests to identify the significant differences.

Supplementary Table 1. Proteins with signal peptides that were negatively affected by TRAP depletion in CDG patient fibroblasts.

Gene	GP %	Hph	signal peptide
ADAM12	17,86	0,82	MAARPLPVSPARALLLALAGALLAPCEA
CDH13	9,09	1,1836	MQPRTPLVLCVLLSQVLLLTSA
CES2	11,54	0,45	MRLHRLRARLSAVACGLLLLLVRGQG
CREG1 *	12,91	0,88	MAGLSRGSARALLAALLASTLLALLVSPARG
CTBS	18,42	0,4616	MSRPQLRRWRLVSSPPSGVPGLALLALLALLALRLAAG
EFNB2	3,7	0,2859	MAVRRDSVWKYCWGVLMVLCRTAISKS
ENG	12	0,9224	MDRGTLPLAVALLLASCSLSPTSLA
F3	18,75	0,0694	METPAWPRVPRPETAVARTLLLGWVFAQVAGA
GPC6	26,08	1,5617	MPSWIGAVILPLLGLLLSLPAGA
HEG1	31,04	0,5793	MASPRASRWPPPLLLLLLPLLLLPPAAPG
HLA-B:P30462	4,17	1,2933	MLVMAPRTVLLLLSAALALTETWA
HLA-B:P30685	8,34	0,76	MRVTAPRTVLLLLWGAVALTETWA
HSPG2	14,29	1,0486	MGWRAAGALLLALLLHGRLLA
LAMA2	31,82	1,3764	MPGAAGVLLLLLLSGGLGGVQA
LOXL2	8	1,108	MERPLCSHLCSCLAMLALLSPLSLA
NEO1	15,15	0,1097	MAAERGARRLLSTPSFWLYCLLLLGRRAPGAAA
NOMO1	32,26	1,0755	MLVGQGAGPLGPAVVTAAVVLLLSGVGPAHG
PCDH17	5,88	1,72	MYLSICCCFLLWAPALT
PLA2G15	21,21	1,0539	MGLHLRPYRVGLLPDGLLFLLLLLMLLADPALP
PODN	10,53	1,1284	MAQSRVLLLLLLLPPQLHL
RNASET2	20,84	1,2767	MRPAALRGALLGCLCLALLCLGGA
SEMA7A	29,55	-0,0541	MTPPPPGRAAPSAPRARVPGPPARLGLPLRLRLLLLLWAAAASA
SERPINE2	10,53	1,2284	MNWHLPLFLLASVTLPSIC
SSR2/TRAPB *	0	1,6835	MRLLSFVVLALFAVTQA
SSR4/TRAPD *	4,35	1,3591	MAAMASLGALALLLLSSLSRCSA
SULF1	9,09	1,4186	MKYSCCALVLAVLGTELLGSLC
TAPBP	5	1,5825	MKSLSLLLAVALGLATAVSA
TMCO3	13,64	0,9977	MKVLGRSFFWVLFPVLPWAVQA
TMED1	21,74	1,3183	MMAAGAALALALWLLMPPVEVGG
TNFRSF11B	0	0,8786	MNNLLCCALVFLDISIKWTTQ
TUSC3 */***	24,39	-0,0107	MGARGAPSRRRQAGRRLRYLPTGSFPFLLLLLLLCIQLGGG
TWSG1 *	4	1,2692	MKLHYVAVLTLAILMFLTWLPESLS
TXNDC15	25	0,2838	MVPAAGRRPPRVMRLLGWWQVLLWVLGLPVRG
EPDR1 *	29,73	0,727	MPGRAPLRTVPGALGAWLLGGLWAWTLCGLCSLGAVG

Amino acid sequences of signal peptides (SP) are shown together with gene name, GP content in %, and hydrophobicity (Hph). *, proteins, which were also negatively affected by TRAP depletion in HeLa cells; ***, OST subunits. The *DDOST* gene codes for Ost48.

Supplementary Table 2. Proteins with N-terminal transmembrane helices that were negatively affected by TRAP depletion in CDG patient fibroblasts.

Gene	GP %	Hph	N-terminal transmembrane helix
ARMC10	21,74	0,78	RGAGWVAAGLLLGAGACYCIYRL
ARMCX6	19,05	1,1414	VGWMAAGLMIGAGACYCVYKL
ATP13A3	14,29	2,1771	LAIVSLGVICSGGFLLLLLYW
ATP1A2	14,29	0,8176	EWVKFCRQLFGGFSILLWIGA
ATP2A3	0	1,2314	LWELVLEQFEDLLVRILLLAA
C17orf62	17,39	1,0209	GIRSWSLLVGILSIGLAAAYYSG
C1GALT1	8,7	1,6713	LNFLTFLCGSAIGFLLCSQLFSI
CDIPT	9,52	1,8552	IFLFVPNLIGYARIVFAIISF
CHPF2	17,4	1,7096	LALLRPALPLILGLSLGCSLSLL
CHRM2	8,7	2,5096	VVFIVLVAGSLSLVTIIGNILVM
CHST14	9,52	2,2314	LLPSMLMFAVIVASSGLLLMI
EXT2	9,52	1,699	YITLFSIVLLGLIATGMFQFW
FAM20B	0	3,0784	VVLLAILLVIFIFTKVFLI
JPH2	4,76	2,7838	ILICMVILLNIGLAILFVHLL
KCNJ2	4	2,1992	RWMLVIFCLAFVLSWLFFGCVFWLI
LMBRD2	6,25	2,76	LGLEIVFVFFLALFLL
MFSD10	14,28	2,2933	VVFLGLLLDLLAFTLLLPLLP
NOC4L	14,29	1,499	ACDLGGALSLLALNGLFILIH
PEX13	16	1,1516	AATSAKSWPIFLFFAVILGGPYLIW
PIGG	4,76	2,1219	IYSMMVGTVVVLEVLTLLLLS
POM121C;POM121	14,28	1,9457	LVGLLLYLVPAAAALAWLAVG
SGMS2	4,76	2,1076	GIAFIYAVFNLVLTTVMITVV
SGPP1	14,28	1,1505	FCFGTELGNELFYILFFPFWI
SLC22A18	0	1,601	ILLTYVLAATELTCLFMQFSI
SLC2A10	23,81	1,4352	LLGGLTFGYELAVISGALLPL
SLC38A10	9,52	1,0933	AAASNWGLITNIVNSIVGVSV
SLC44A2	9,52	2,82	IICCVFLLLAIVGYVAVGIIA
SLC7A14	23,81	1,6476	LISLGVGSCVGTGMYVVSGLV
SLC9A6	28,57	0,821	LWLLLAVGVFDWAGASDGGGG
SSR3/TRAPG *	9,52	1,2971	SSALFFGNAFIVSAIPIWLYW
STX17	38,09	1,4886	LAALPVAGALIGGMVGGPIGL
TENM3	4,76	2,3457	ALCAVGVSVLLAILLSYFIAM
TMEM159	9,52	2,9314	LLVFIVMSAVPVGFFLLIVVL
TMEM168;FLJ13576	9,52	1,279	LGYLARINLLVAICLGLYVRW
TMEM189	0	1,9781	WCSVILCFSLIAHNLVHLLLL
TMEM199	0	2,3	LVITIFNFIVTVVAAFVCTYL
TMEM261	27,78	1,1856	VLSGLGLMGAGGYVYWVA
TMEM39A	0	2,1724	SLLFEFLFFIYLLVALFIQYI
TMEM55B	14,29	2,1229	CICCFLLGLLLAVTATGLAFG
TPST2	11,76	2,1965	LLAAGCALVLVLAVQLG
TSPAN10	14,28	2,0152	LIFLSNFPFSLLGLLALAIGL

Amino acid sequences of N-terminal transmembrane helices (TMH) are shown, along with the protein accession number, gene name, presence of N-glycosylation sites (N-glyc), GP content in %, and hydrophobicity (Hph). Transmembrane helices were identified according to the TMHMM server 2.0 (www.cbs.dtu.dk/services/TMHMM/). *, proteins, which were also negatively affected by TRAP depletion in HeLa cells.

Supplementary Table 3. Sequences of siRNAs used in this study.

name	target gene	sequence
Sec61A1-siRNA	*SEC61A1*	GGAAUUUGCCUGCUAAUCAdTdT
Sec61A1-UTR-siRNA	*SEC61A1*	CACUGAAAUGUCUACGUUUdTdT
TRAPA-siRNA	*TRAPA*	AGAUUUGAACGGCAAUGUAdTdT
TRAPB-siRNA	*TRAPB*	CCUCGGCAACAAUUACUUAdTdT
TRAPB-UTR-siRNA	*TRAPB*	AAGGGUAUCUUAAAUGCAAdTdT

Supplementary Table 4. Sequences of primers used in this study.

Gene (size of PCR product)		Sense Primer (5' --- 3')	Antisense Primer (5' --- 3')
PPIC (142 bp)	signal peptide	GGATCCGGTACCGAG GAGATCT	GGCAAGCTTGCCCTC GGCCCCCGAAGAAAA CACAA
PPIB (5396 bp)	mature part	TGAAAGCTTGATGAGA AGAAGAAGGGGCCC	CAGCAGCAGGAAGAA GACGGAC
PPIC-PPIB (5532 bp)	linker removal	GTTTTCTTCGGGGGC CGAGGGCGATGAGAA GAAGAAGGGGCCCAA AG	CTTTGGGCCCCTTCTT CTTCTCATCGCCCTCG GCCCCCGAAGAAAAC
TMED5 (5460 bp)	PG23/24AA	CTTCTGGCCGCTCTG CCTCCGGTGCTGCTG GCAGCAGCGGCCGGC TTCACACCTTCCCTCG ATAGC	GCTATCGAGGGAAGG TGTGAAGCCGGCCGC TGCTGCCAGCAGCAC CGGAGGCAGAGCGGC CAGAAG
PPIB a) (5529 bp) b) (5529 bp)	a) SE5/6GP	GATCGCCATGCTGCG CCTCGGACCACGCAA CATGAAGGTGCTCCTT G	CAAGGAGCACCTTCAT GTTGCGTGGTCCGAG GCGCAGCATGGCGAT C
	b) AA14/15PP	GACCACGCAACATGA AGGTGCTCCTTCCACC AGCCCTCATCGCGGG GTCCGTCTTCTTC	GAAGAAGACGGACCC CGCGATGAGGGCTGG TGGAAGGAGCACCTT CATGTTGCGTGGTC

Supplementary Note 1 |Validation of the used imputation method. We assumed that the reason for missing values is that they stem from "the bottom" of the distribution and belong to low abundance proteins that were not detected by the mass spectrometry instrument. Here, we present a systematic analysis to what extent the data imputation may affect the differential abundance analysis. For this, we assumed that the missing values stem either from the bottom 5th percentile or 10th percentile of the distribution. The first Sec61 silencing experiment was selected for the validation. Out of all protein entries, we selected only those proteins that have a "complete" dataset, i.e. none of out of nine entries was missing. For this dataset, this was the case for 5715 out of 6960 proteins. For simplicity, all protein intensities were converted into log2 values. To generate a synthetic dataset for missing data, we randomly removed 10% of the (known) data points from the lower tail of the distribution below a certain threshold. In other words, out of, say, 100 values that are below the threshold, 10 values were randomly removed. We tested two different thresholds (5th and 10th percentile of the overall distribution). For both thresholds, we repeated the removal 100 times. Therefore, in total, we generated 200 new datasets with artificially generated "missing" data. Subsequently, these "missing" data points were imputed using the imputation method described in the Methods section. Then, a differential protein abundance analysis was carried out on the imputed and the original data as described in the Methods section. Finally, we compared the results of the differential analysis of the imputed and original data to validate the reliability of the imputation method. For this, using the results of the previous steps, the significantly affected proteins were either labelled as 1 (positively affected) or as -1 (negatively affected) while the unaffected proteins were labelled 0. Afterwards, we computed the Pearson correlation coefficient between the results of the original data and of the imputed data. The overall correlation coefficients for the 5th and 10th percentile thresholds are 0.975 ± 0.018 and 0.927 ± 0.020, respectively. Thus this validation shows that the imputation method is very reliable since the results of the original data and the imputed data are highly correlated.

Supplementary References

1. Garnier, J., Osguthorpe, D. J. & Robson, B. Analysis of the accuracy and implications of simple methods for predicting the secondary structure of globular proteins. *J. Mol. Biol.* **120**, 97-120 (1978).
2. Kyte, J. & Doolittle, R. F. A simple method for displaying the hydrophobic character of a protein. *J. Mol. Biol.* **157**, 105-132 (1982).
