# Supplementary material for: Proteomics reveals signal peptide features determining the client specificity in human TRAP-dependent ER protein import
Source: Nat Commun. 2018 Sep 14;9:3765. doi: 10.1038/s41467-018-06188-z (PMC6138672; doi:10.1038/s41467-018-06188-z)
Supplement: Supplementary file 3 — Description of Additional Supplementary Files [file 41467_2018_6188_MOESM3_ESM.pdf]

Description of Additional Supplementary Files

File Name: Supplementary Data 1
Description: Alphabetical list of genes corresponding to proteins quantified in both Sec61 depletion experiments. Gene names, protein accession numbers, and log2 fold changes resulting from Sec61 depletion are indicated. Minus sign denotes negatively affected proteins. The number of listed proteins differs from the total number of quantified proteins because some proteins were not quantified in both experiments, or were quantified in less than two of the triplicates in at least one experiment. The original Orbitrap data for all quantified proteins are deposited at Proteome Exchange: http://www.proteomexchange.org

File Name: Supplementary Data 2.
Description: Alphabetical list of genes corresponding to proteins quantified in all three TRAP depletion experiments. Gene names, protein accession numbers, and log2 fold changes resulting from TRAP depletion are indicated. Minus sign denotes negatively affected proteins. The number of proteins listed here differs from the total number of quantified proteins because some proteins were not quantified in all three experiments or were quantified in less than two of the triplicates in at least one experiment.

File Name: Supplementary Data 3.
Description: Proteins that were negatively affected by Sec61 depletion, i.e. potential Sec61 clients. Gene names, protein accession numbers, and log2 fold changes resulting from Sec61 depletion are presented together with Gene Ontology (GO) annotations for subcellular location(s), presence of sp or tmh, and number of N-glycosylation sites, as extracted from UniProtKB entries using custom scripts. Proteins are listed according to decreasing negative effects of Sec61 depletion. Sec61α and Sec61β are located at positions 15 and 92, respectively.

File Name: Supplementary Data 4.
Description: Proteins that were positively affected by Sec61 depletion. Gene names, protein accession numbers, and log2 fold changes resulting from Sec61 depletion are presented together with Gene Ontology (GO) annotations for subcellular location(s), presence of sp or tmh, and the number of N-glycosylation sites, as extracted from UniProtKB entries using custom scripts. Proteins are listed according to decreasing positive effects of Sec61 depletion. The SRP receptor subunit β is located at position 88, while SRP receptor subunit α is not listed here because it was not significantly affected in one of the two experiments (Supplementary Fig. 1c).

File Name: Supplementary Data 5.
Description: Proteins that were negatively affected by TRAP depletion, i.e. potential TRAP clients. Gene names, protein accession numbers, and log2 fold changes resulting from TRAP depletion are given together with Gene Ontology (GO) annotations for subcellular location(s), presence of sp or tmh, and the number of N-glycosylation sites, as extracted from UniProtKB entries using custom scripts. 4 4 Proteins are listed according to decreasing negative effects of TRAP depletion. TRAPα, TRAPβ, TRAPγ, and TRAPδ are listed at positions 6, 4, 2, and 3, respectively.

File Name: Supplementary Data 6.
Description: Proteins that were positively affected by TRAP depletion. Gene names, protein accession numbers, and log2 fold changes resulting from TRAP depletion are presented together with Gene Ontology (GO) annotations for subcellular location(s), presence of sp or tmh, and the number of N-glycosylation sites, as extracted from UniProtKB entries using custom scripts. Proteins are listed according to decreasing positive effects of TRAP depletion. SRP receptor subunits α and β are listed at positions 63 and 34, respectively.

File Name: Supplementary Data 7.
Description: Summary of significant negative and positive changes after Sec61 or TRAP depletion. Protein accession numbers, gene names, and full protein names are presented for all proteins that were significantly changed after TRAP or Sec61 depletion to highlight the overlaps between the two depletions. Changes are shown in binary code, with 1 denoting a significant effect.

File Name: Supplementary Data 8.
Description: Proteins that were negatively affected by TRAP depletion in CDG patient fibroblasts. Gene names and protein accession numbers resulting from TRAP depletion in CDG patient fibroblasts are given together with Gene Ontology (GO) annotations for subcellular location(s), presence of sp or tmh, and the number of N-glycosylation sites, as extracted from UniProtKB entries using custom scripts. Proteins are listed according to decreasing negative effects of TRAP depletion. We refrained from giving log2 fold changes because of the low n of these analyses.

File Name: Supplementary Data 9.
Description: Proteins that were positively affected by TRAP depletion in CDG patient fibroblasts.
